# DNA-binding protein PfAP2-P regulates parasite pathogenesis during malaria parasite blood stages

**DOI:** 10.1038/s41564-023-01497-6

**Published:** 2023-10-26

**Authors:** Amit Kumar Subudhi, Judith L. Green, Rohit Satyam, Rahul P. Salunke, Todd Lenz, Muhammad Shuaib, Ioannis Isaioglou, Steven Abel, Mohit Gupta, Luke Esau, Tobias Mourier, Raushan Nugmanova, Sara Mfarrej, Rupali Shivapurkar, Zenaida Stead, Fathia Ben Rached, Yogesh Ostwal, Rachid Sougrat, Ashraf Dada, Abdullah Fuaad Kadamany, Wolfgang Fischle, Jasmeen Merzaban, Ellen Knuepfer, David J. P. Ferguson, Ishaan Gupta, Karine G. Le Roch, Anthony A. Holder, Arnab Pain

**Affiliations:** 1 Pathogen Genomics Group, Bioscience Program, Biological and Environmental Science and Engineering (BESE) Division, King Abdullah University of Science and Technology, Thuwal, Kingdom of Saudi Arabia; 2 Malaria Parasitology Laboratory, The Francis Crick Institute, London, UK; 3 Department of Computer Science, Jamia Millia Islamia, New Delhi, India; 4 Department of Molecular, Cell and Systems Biology, University of California Riverside, Riverside, CA USA; 5 Cell Migration and Signaling Laboratory, Bioscience Program, BESE Division, King Abdullah University of Science and Technology, Thuwal, Kingdom of Saudi Arabia; 6 KAUST Core Labs, King Abdullah University of Science and Technology, Thuwal, Kingdom of Saudi Arabia; 7 Laboratory of Chromatin Biochemistry, Bioscience Program, BESE Division, King Abdullah University of Science and Technology, Thuwal, Kingdom of Saudi Arabia; 8 Department of Pathology and Laboratory Medicine, King Faisal Specialist Hospital and Research Center, Jeddah, Kingdom of Saudi Arabia; 9 College of Medicine, Al Faisal University, Riyadh, Saudi Arabia; 10 Nuffield Department of Clinical Laboratory Science, University of Oxford, John Radcliffe Hospital, Oxford, UK; 11 Department of Biological and Medical Sciences, Faculty of Health and Life Sciences, Oxford Brookes University, Oxford, UK; 12 Department of Biochemical Engineering and Biotechnology, Indian Institute of Technology Delhi, New Delhi, India; 13 School of Artificial Intelligence, Indian Institute of Technology Delhi, New Delhi, India; 14 International Institute for Zoonosis Control, Hokkaido University, Sapporo, Japan; 15 Present Address: Molecular and Cellular Parasitology Laboratory, Department of Pathobiology and Population Sciences, The Royal Veterinary College, Hatfield, UK

**Keywords:** Cellular microbiology, Gene silencing

## Abstract

Malaria-associated pathogenesis such as parasite invasion, egress, host cell remodelling and antigenic variation requires concerted action by many proteins, but the molecular regulation is poorly understood. Here we have characterized an essential *Plasmodium*-specific Apicomplexan AP2 transcription factor in *Plasmodium falciparum* (PfAP2-P; pathogenesis) during the blood-stage development with two peaks of expression. An inducible knockout of gene function showed that PfAP2-P is essential for trophozoite development, and critical for *var* gene regulation, merozoite development and parasite egress. Chromatin immunoprecipitation sequencing data collected at timepoints matching the two peaks of *pfap2-p* expression demonstrate PfAP2-P binding to promoters of genes controlling trophozoite development, host cell remodelling, antigenic variation and pathogenicity. Single-cell RNA sequencing and fluorescence-activated cell sorting revealed de-repression of most *var* genes in *Δpfap2-p* parasites. *Δpfap2-p* parasites also overexpress early gametocyte marker genes, indicating a regulatory role in sexual stage conversion. We conclude that PfAP2-P is an essential upstream transcriptional regulator at two distinct stages of the intra-erythrocytic development cycle.

## Abstract (web summary)

The *Plasmodium falciparum* transcription factor PfAP2-P is an upstream regulator of parasite blood-stage development and regulates trophozoite development, host cell remodelling, antigenic variation and pathogenicity.

## Main

Regulation of gene expression involves sequence-specific DNA-binding proteins as transcriptional activators or repressors and epigenetic modifiers^1^. Chromatin-mediated regulation of *Plasmodium falciparum* gene expression controls invasion proteins and antigenically variant proteins^2^. Recruitment of epigenetic regulators is poorly understood, but may involve DNA-binding regulatory proteins. In the genus *Plasmodium*, a family of 26 (27 in *P. falciparum*) Apicomplexan-specific AP2 (ApiAP2) DNA-binding proteins has been identified as the major transcriptional regulator of processes during development, differentiation and response to environmental changes^3–8^. However, the detailed functions of only a few have been characterized and the role of the remainder is unknown.

In this Article, we use inducible knockout of gene function^9,10^ to show that PF3D7_1107800 codes for a *Plasmodium*-specific ApiAP2 in *P. falciparum* (PfAP2-P) that is essential for cell cycle, antigenic variation and parasite egress and invasion. We establish that PfAP2-P directly or indirectly regulates the expression of genes involved in host cell remodelling, antigenic variation, egress, invasion and gametocytogenesis among the pathogenesis-related processes corresponding to the two peaks of expression during the intra-erythrocytic development cycle (IDC).

## Results

### PfAP2-P is essential for parasite development and growth

**Extended Data Fig. 1:**
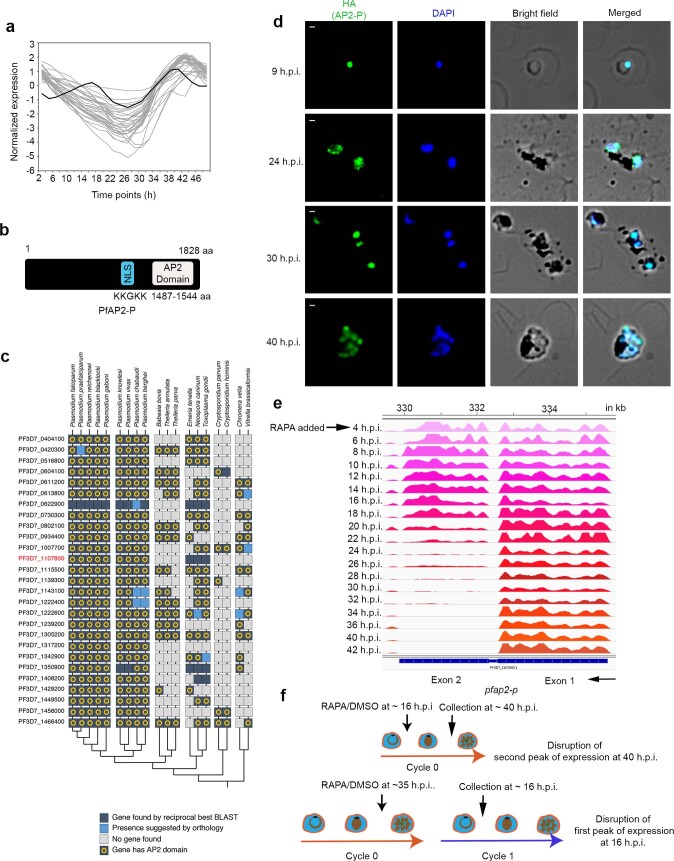
Features of PfAP2-P DNA binding protein. **a**, Expression of *pfap2-p* (thick black line) and the top 40 down-regulated genes (grey lines) in *P. falciparum* line II3 over a 48-hour IDC^14^. **b**, The primary structure of PfAP2-P, with a nuclear localization signal (NLS) and a single AP2 DNA binding domain. Both NLS and DNA binding domain are encoded by exon 2 of the gene. **c**, A phylogenetic distribution of AP2 genes in the genus *Plasmodium* and related alveolates. **d**, IFA shows that PfAP2-P (HA tagged) localizes to the parasite nucleus at various IDC developmental stages (representative of two independent experiments). Scale bar 2μM. **e**, RNA-seq data from different IDC time-points mapped to the *pfap2-p* locus; there is a drastic reduction in RNA-seq reads mapping to the second exon 16 hours after the addition of rapamycin that is at 20 h.p.i. **f**, Schematic showing rapamycin treatment schedule to disrupt either first or second peak of *pfap2-p* expression. Panel f created with Biorender.com.

In a recent study^11^, we identified 363 genes with a 24 h (circadian-like) rhythmic periodicity of expression in the 48-h *P. falciparum* IDC, one of which was an ApiAP2, now designated as PfAP2-P (Extended Data Fig. 1a). The first peak of expression at ~16 h post-invasion (h.p.i.) coincides with maximal *var* gene family expression. The second peak at ~40 h.p.i. is just before maximal expression of known invasion- and egress-associated genes, suggesting PfAP2-P had a role in their regulation (Extended Data Fig. 1a).

**Fig. 1:**
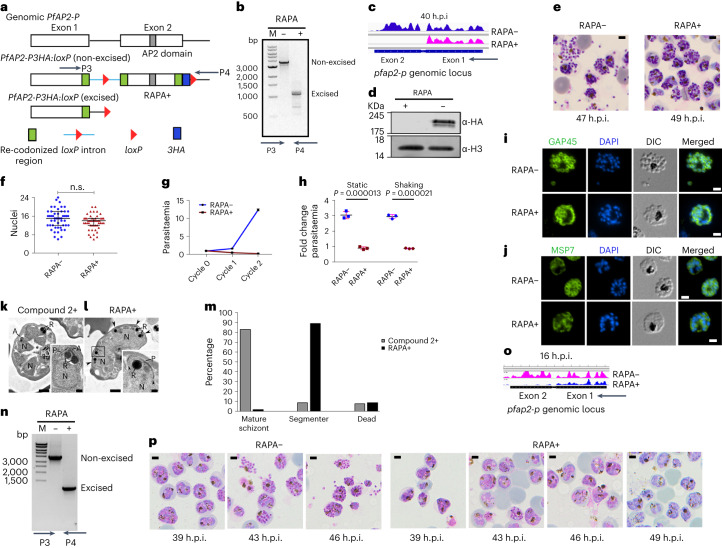
PfAP2-P is essential for parasite growth and development. **a**, Schematic of strategy showing conditional truncation by excision of the *loxP-*flanked second exon. **b**, Confirmation of exon 2 excision in RAPA-treated parasites. **c**, RNA-seq reads coverage from 40 h.p.i. parasites of *pfap2-p* locus. **d**, Western blot of control (RAPA−) and rapamycin (RAPA+)-treated PfAP2-P-3HA:*loxP* schizont extracts probed with anti-HA and anti-histone H3 antibodies. **e**, Images of Giemsa-stained (control, RAPA− and treated, RAPA+; treatment at 16 h.p.i.) schizonts at end of cycle 0 (representative of four independent experiments). Scale bar, 2 μm. **f**, Nuclei in control (RAPA−) and treated (RAPA+) schizonts (*n* = 50). *P* value of 0.178, one-way ANOVA using Šidák’s multiple comparisons test, assumed Gaussian distribution; error bar is mean with ± standard deviation (s.d.). n.s., not significant. **g**, Replication of control (RAPA−) and treated (RAPA+) parasites over two growth cycles (three biological replicates, average parasitaemia ± s.d.). **h**, Erythrocyte invasion by control (RAPA−) and treated (RAPA+) PfAP2-P-3HA:*loxP* parasites under static and shaking conditions. Statistical significance: two-tailed *t*-test; RAPA− versus RAPA+ parasites in static conditions (*n* = 3, *t* = 14.17, degrees of freedom 4, *P* = 0.000013, 95% confidence interval 1.606–2.389) and RAPA– versus RAPA+ parasites in shaking conditions (*n* = 3, *t* = 12.41, degrees of freedom 4, *P* = 0.000021, 95% confidence interval 1.578–2.487). Error bars are means ± s.d. **i**,**j**, Immunofluorescence microscopy of control (RAPA−) and treated (RAPA+) parasites showing distribution of GAP45 (**i**) and MSP7 (**j**). Scale bar, 2 µm. DIC, differential interference contrast. **k**,**l**, Electron micrographs of iRBCs treated with compound 2 (**k**) or rapamycin (RAPA+) (**l**); R, rhoptries; FD, food vacuole; A, apical polar ring; N, nucleus; P, pellicle; scale bar, 1 µm or 100 nm (inserts). **m**, The different developmental stages of compound 2-treated and RAPA-treated parasites (at 49 h.p.i.). **n**, Confirmation of exon 2 excision in RAPA-treated parasites at 16 h.p.i. **o**, RNA-seq reads coverage from 16 h.p.i. parasites (cycle 1) of *pfap2-p* locus. **p**, Images of Giemsa-stained parasites in cycle 1 following parasite treatment with DMSO (RAPA−) or rapamycin (RAPA+) at 35 h.p.i. in cycle 0. Scale bar, 2 μm. Results in **b**–**d** are representative of three independent experiments and results in **i**–**l** and **n**–**p** are representative of two independent experiments. DAPI, 4′,6-diamidino-2-phenylindole. Source data

PfAP2-P has one AP2 domain (residues 1,487–1,544, PFAM ID: PF00847) and a nuclear localization signal, encoded in the second exon (Extended Data Fig. 1b). PfAP2-P orthologues are unique to *Plasmodium* spp. (Extended Data Fig. 1c), and PfAP2-P and its *Plasmodium berghei* orthologue (PBANKA_0939100) are essential for blood-stage development^12,13^. To investigate the function of PfAP2-P, we generated a triple human influenza hemagglutinin (3HA)-tagged PfAP2-P inducible exon 2 knockout of *P. falciparum* (PfAP2-P-3HA:loxP) in a parental line expressing rapamycin (RAPA)-inducible dimerizable Cre recombinase (DiCre)^10^ (Fig. 1a and Methods). Immunofluorescence assays (IFA) confirmed its nuclear location at various stages suggesting a basal level of PfAP2-P protein throughout the IDC (Extended Data Fig. 1d).

Following RAPA addition to synchronized parasite culture, the *loxP*-flanked *pfap2-p* second exon was efficiently excised (Fig. 1b). A time-series RNA sequencing (RNA-seq) analysis revealed that deletion and disruption of *pfap2-p* expression occurred ~16 h after RAPA addition (Extended Data Fig. 1e); therefore, to ablate only the second peak of functional *pfap2-p* expression at 40 h.p.i. in cycle 0, RAPA was added at ~16 h.p.i in the same cycle (that is cycle 0) and to ablate the first peak of expression at 16 h.p.i. in cycle 1, RAPA was added at ~35 h.p.i. in cycle 0, with parasite collection at 16 h.p.i. in cycle 1 (Extended Data Fig. 1f). Henceforth, *Δpfap2-p-*16 refers to parasites collected at 16 h.p.i. with exon 2 deleted before the first peak of expression and *Δpfap2-p-*40 refers to parasites collected at 40 h.p.i. with exon 2 deleted before the second peak of expression.

RAPA addition at ~16 h.p.i in cycle 0 ablated the second peak in the same cycle, shown by RNA-seq (Fig. 1c) and western blot (Fig. 1d), and these parasites progressed to morphologically normal segmented schizonts (49 h.p.i, cycle 0; Fig. 1e), with no statistically significant difference in the number of nuclei in late schizonts of *Δpfap2-p* and dimethylsulfoxide (DMSO) (mock)-treated PfAP2-P-3HA:loxP parasites (Fig. 1f). However, *Δpfap2-p* parasites failed to egress (Fig. 1e), with a resultant dramatic reduction in parasitaemia compared with controls (Fig. 1g), indicating an essential role of PfAP2-P in parasite proliferation. In assays under either static or vigorous shaking (shear) conditions with schizonts, no egress or increased parasitaemia was observed for *Δpfap2-p* parasites in contrast to control cultures irrespective of conditions (Fig. 1h); therefore, mechanical disruption of the infected red blood cell (iRBC) does not facilitate *Δpfap2-p* parasite egress.

**Extended Data Fig. 2:**
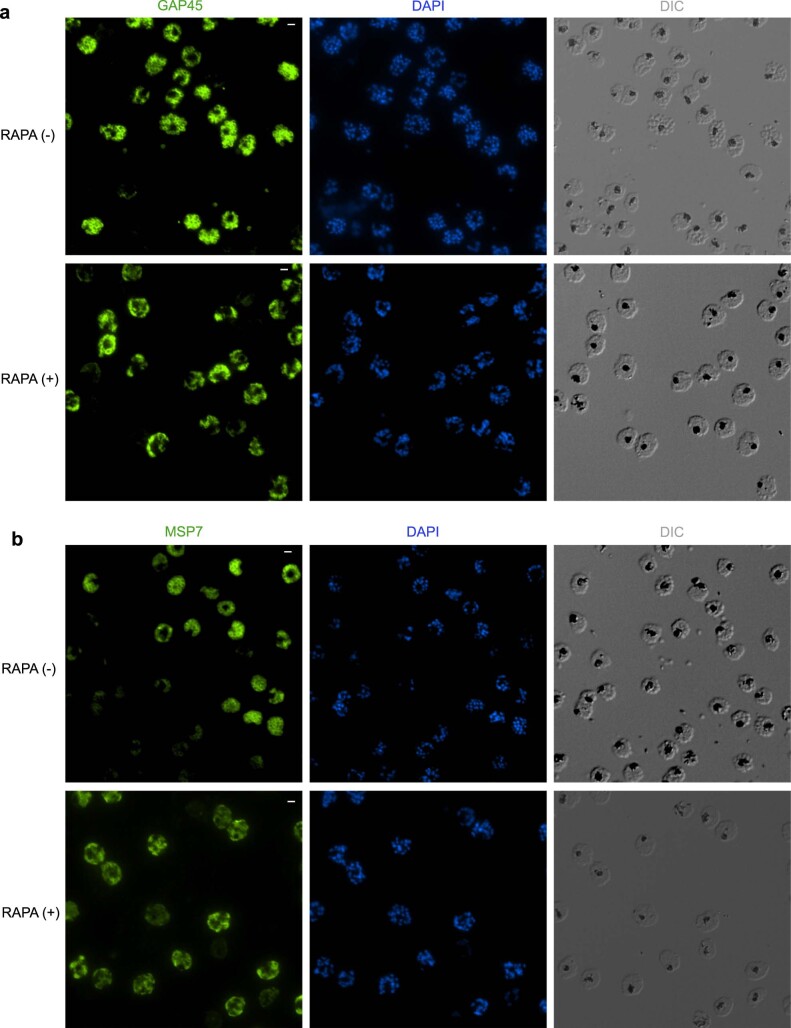
Truncation of *pfap2-p* affects merozoite development. **a**, Uncropped images of figure presented as Fig. 1i. **b**, Uncropped images of figure presented as Fig. 1j. Scale bar 2μM.

To discern morphological differences, we compared *Δpfap2-p* and control parasites by IFA with antibodies specific for parasite surface pellicle proteins: namely GAP45, a protein of the glideosome/inner membrane complex, and merozoite surface protein 7 (MSP7) (Fig. 1i,j and Extended Data Fig. 2a,b). In *Δpfap2-p* parasites compared with controls, the distribution of these proteins was disordered. Electron microscopy analysis of *Δpfap2-p* and parasites treated with compound 2 (an inhibitor of egress^14^) at 49 h.p.i. revealed that most mature schizonts contained fully formed merozoites in the control sample (Fig. 1k and Fig. 1k, insert), but most *Δpfap2-p* schizonts were observed at an earlier segmented stage (Fig. 1l and Fig. 1l, insert). In 50 iRBC samples, the control and *Δpfap2-p* groups contained 83% and 2% mature schizonts, respectively (Fig. 1m). These data suggest that PfAP2-P function after the second expression peak is manifested after nuclear division in the final IDC stages.

RAPA treatment from ~35 h.p.i. in cycle 0 had little effect on parasite egress or invasion, but functional deletion of *pfap2-p* occurred in the next cycle before the first expression peak. *pfap2-p*-16 parasites were collected (Extended Data Fig. 1f), and excision of exon 2 and loss of functional PfAP2-P expression were confirmed by RNA-seq (Fig. 1n,o). Parasite development stalled at the late trophozoite/early schizont stage in cycle 1 (Fig. 1p), suggesting that *pfap2-p* expression plays a critical role in parasite development immediately after its first peak at ~16 h.p.i.

### PfAP2-P is a crucial regulator of malaria pathogenesis-associated genes

**Extended Data Fig. 3:**
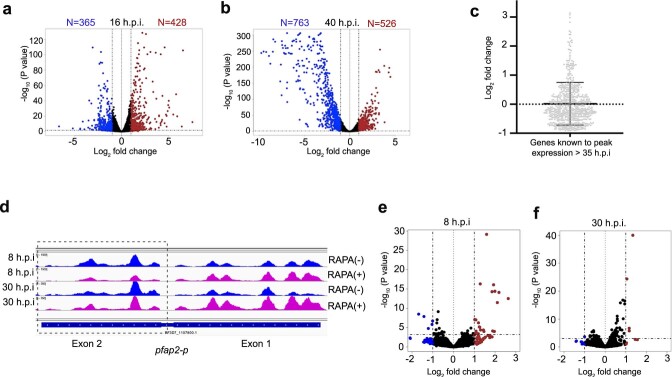
Truncation of *pfap2-p* affects parasite’s transcriptome. **a, b**, Volcano plots showing significantly differentially expressed genes in rapamycin-treated compared to control parasites at 16 h.p.i. (**a**) and 40 h.p.i. (**b**). **c**, non-differentially expressed genes and up-regulated genes (n = 658) in treated [RAPA (+)] compared to control [RAPA (-)] parasites at 40 h.p.i., which had been reported to express at least 4-fold higher in schizont-stage parasites (>35 h.p.i.) compared to early-stage parasites (<35 h.p.i.)^5^; error bar is mean with ± s.d. **d**, RNA-seq reads coverage from 8 and 40 h.p.i. parasites of *pf-ap2-p* locus. **e-f** Volcano plots showing significantly differentially expressed genes in rapamycin-treated compared to control parasites at 8 h.p.i. (**e**) and 30 h.p.i. (**f**).

Comparative RNA-seq analysis of *Δpfap2-p* parasites and controls at 16 and 40 h.p.i. identified 793 and 1,289 differentially expressed genes (false discovery rate (FDR) ≤0.05), respectively (Extended Data Fig. 3a,b and Supplementary Data 1). Disruption of the second expression peak caused parasites to stall at the late segmented stage, so we examined whether these transcriptional differences at 40 h.p.i. were directly due to the deletion of exon 2 or to reduced viability. We focused on 1,042 genes that are known to have peak expression after 35 h.p.i.^1^, expecting that death or developmental delay of *Δpfap2-p-*40 parasites would result in most of these genes being down-regulated. However, 658 (63%) had no significant change in expression level (Extended Data Fig. 3c), indicating that the observed gene expression differences were directly due to *pfap2-p* deletion and not reduced viability.

Time-series RNA-seq data (Extended Data Fig. 1e) showed that it takes 16–20 h after RAPA treatment to delete the loxP-flanked sequences from most parasites, with majority of the deletion taking place in the last 2–3 h. To assess whether RAPA addition substantially affected gene expression well before transcriptome analysis at 16 and 40 h.p.i., we performed RNA-seq analysis on parasites collected at 8 h.p.i. in cycle 1 following RAPA addition at 35 h.p.i in cycle 0 and at 30 h.p.i after adding RAPA at 16 h.p.i in the same cycle (stages before maximum expression of *pfap2-p*). We observed a slight reduction of exon 2 reads in RAPA-treated parasites compared with mock-treated parasites (Extended Data Fig. 3d). Only 88 genes were differentially expressed at 8 h.p.i. compared with the 763 genes at 16 h.p.i., and 17 genes were differentially expressed at 30 h.p.i. compared with 1,289 genes at 40 h.p.i. (Extended Data Fig. 3e,f and Supplementary Data 1). These data show that, even after 10–12 h, RAPA addition has no strong effect on transcription, probably because *pfap2-p* truncation has yet to occur in most parasites.

**Fig. 2:**
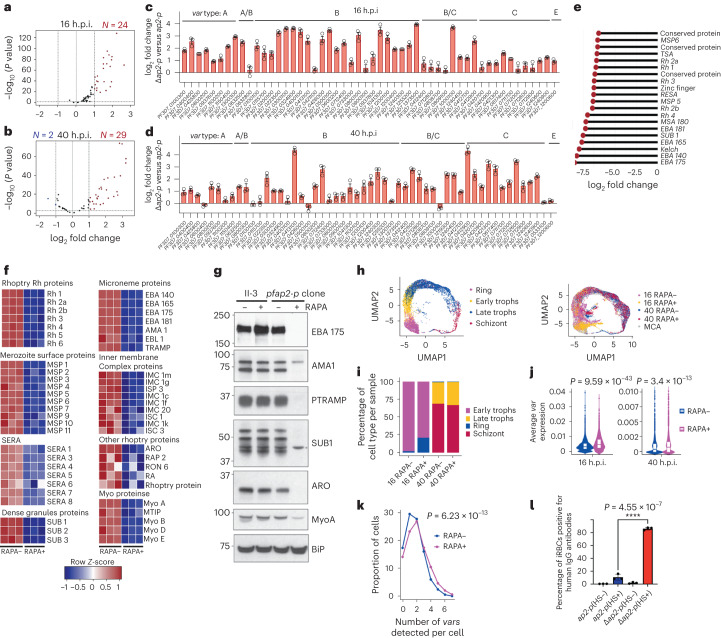
PfAP2-P regulates most of the malaria pathogenesis-associated genes. **a**,**b**, Volcano plots showing differentially expressed *var* genes in 16 h.p.i (cycle 1) (**a**) and 40 h.p.i. (cycle 0) (**b**) *∆pfap2-p* parasites. **c**,**d**, Differential expression of *var* genes (*n* = 3 biological replicates) measured by qRT–PCR at 16 h.p.i. (**c**) and 40 h.p.i. (**d**); error bar is standard error of the mean. **e**, Lollipop plot of expression level of top 20 significantly down-regulated genes in ∆*pfap2-p* compared with control parasites at 40 h.p.i. **f**, Heatmaps of down-regulated known egress and invasion genes in treated (RAPA+) or control (RAPA−) parasites, grouped on the basis of the subcellular location of their products. **g**, Western blots of schizont extract from parental II3 and PfAP2-P-3HA parasites in the absence (−) or presence (+) of rapamycin, probed with antibodies specific for invasion proteins. BiP was detected as a loading control. Molecular mass (kDa) of standards on left side of each panel. A non-specific cross-reacting protein on the SUB1 blot is marked with an asterisk. Representative of two independent experiments. **h**, Left: uniform manifold approximation and projection (UMAP) of scRNA-seq data from MCA, with annotated developmental stages. Right: UMAP projections of scRNA-seq in-house data; each dot represents gene expression data from a single parasite (colours corresponding to h.p.i. and *pfap2-p* truncation status) plotted over MCA data. **i**, Distribution of developmental stages of treated (RAPA+) or control (RAPA−) parasites at 16 and 40 h.p.i. **j**, Violin plots of average *var* gene expression in treated (RAPA+) or control (RAPA−) parasites at both 16 h.p.i. and 40 h.p.i. Violin plot shows the median, Q1–Q3 range (box) and distribution of values (violin). *n* = 3,992; 2,747; 3,629 and 3,591 for 16 and 40 h.p.i. control and RAPA-treated cells, respectively. *P* values were calculated using unpaired two-sample Mann–Whitney Wilcoxon test (two-sided) with continuity correction used. **k**, Proportion of cells expressing one or more *var* genes from treated (RAPA+) or control (RAPA−) cultures (*P* = 6.23 × 10^−13^, two-sided Fisher’s exact test, odds ratio 1.53). **l**, Percentage of iRBCs containing control or *∆pfap2-p* parasites bound by IgG from serum of malaria-infected (HS+) patients or untreated samples (HS−). Significance was determined using a two-tailed *t*-test (*t* = 6.687, degrees of freedom 4, *P* < 0.0001, 95% confidence interval 0.9844–2.382; *n* = 3); error bar is ± standard deviation. Source data

Gene Ontology (GO) enrichment analysis of up-regulated genes in *Δpfap2-p-*16 and *Δpfap2-p-*40 parasites identified pathogenesis as the most enriched biological term (adjusted *P* (*P*_adj_) = 1.23 × 10^−7^ and 6.8 × 10^−10^, respectively; Supplementary Data 2). The *var* gene family contains 59 intact genes that show mutually exclusive expression^15^ and has the most members assigned to this GO term, with significant up-regulation of 24 and 29 *var* genes (*P*_adj_ < 0.05) in *Δpfap2-p* compared with control parasites at 16 h.p.i. and 40 h.p.i., respectively (Fig. 2a,b). Data were validated by quantitative real-time polymerase chain reaction (qRT–PCR) (Fig. 2c,d). These results suggest that PfAP2-P contributes to the repression of most *var* genes.

**Extended Data Fig. 4:**
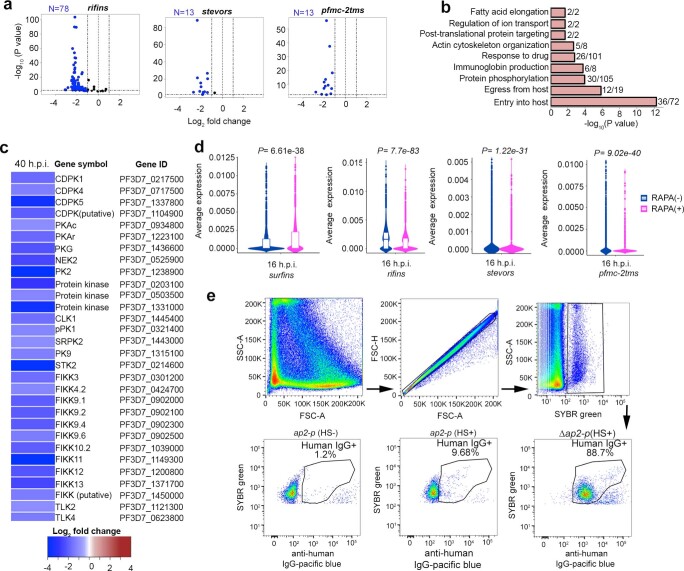
Truncation of *pfap2-p* deregulates malaria pathogenesis-associated genes. **a**, Expression at 16 h.p.i. of members of the gene families: *rifin*, *stevor* and *Pfmc-2tm* that encode antigenically variant proteins. **b**, Gene-ontology (GO) enrichment analysis of all genes down-regulated in rapamycin-treated compared with control parasites at 40 h.p.i. Shown is the number of genes down-regulated in treated parasites out of the total number of genes assigned to that specific GO term. **c**, Heatmap of the expression of all known and putative *P. falciparum* kinases downregulated following rapamycin treatment compared to controls, at 40 h.p.i. **d**, Violin plots of all *surfin*, *rifin, stevor* and *pfmc-2tm* expression per cell in treated [RAPA (+)] and control [RAPA (−)] parasites at 16 h.p.i. Violin plot shows the median, Q1–Q3 (box), distribution of values (violin). n = 3,992, 2,747, 3,629 and 3,591 for 16 and 40 h.p.i. control and RAPA-treated cells respectively. The *P*-values were calculated using unpaired two-samples Mann Whitney Wilcoxon Test (two-sided) with continuity correction was used. **e**, FACS gating strategy for surface PfEMP1 expression detected by IgG binding from pooled serum of malaria-infected individuals. Cells were incubated either without (HS-) or with (HS+) pooled serum from malaria-infected individuals. The percentage of Human IgG+ iRBCs is the mean of three biological replicates. A total of 7,500 Sybr green positive events (iRBCs) per sample were analyzed.

We observed up-regulation in four out of eight *surfins* at 16 h.p.i. (Supplementary Data 1). Gene families coding for other variant proteins such as *rifins* (*n* = 78/132), *stevors* (*n* = 13/30) and *Pfmc-2ms* (*n* = 13/13) were significantly down-regulated (FDR ≤0.05) at 16 h.p.i., suggesting that these genes are positively regulated by PfAP2-P (Extended Data Fig. 4a, Supplementary Data 1). While some *hyp* and *phist* gene family members, encoding exportome-associated proteins, were up-regulated, others were down-regulated at 16 h.p.i., consistent with a functional diversification (Supplementary Data 1). Together, these results suggest that *pfap2-p* expression at 16 h.p.i. has a major role regulating expression of genes coding for proteins important in antigenic variation and host cell remodelling.

GO enrichment analysis of the down-regulated genes in *Δpfap2-p-*40 parasites identified entry into and egress from the host cell as the most enriched biological process terms (Extended Data Fig. 4b and Supplementary Data 2), consistent with the effect of *pfap2-p* deletion on late merozoite development and egress. Of the 20 most down-regulated genes, 15 function in parasite egress or invasion (Fig. 2e). Maximum expression of all most down-regulated genes was just after the second peak of *pfap2-p* expression (Extended Data Fig. 1a), consistent with the proposed role of PfAP2-P in their control. Strikingly, 36 out of 72 known invasion-associated genes, including those coding for micronemal, rhoptry, merozoite surface, inner membrane complex and motor proteins, were down-regulated (Fig. 2f, Supplementary Data 1), and western blot and IFA confirmed the reduced abundance of some of these proteins (Fig. 2g). Altogether, the data suggest that PfAP2-P is essential for egress and invasion-associated gene expression.

Other biological processes enriched in the down-regulated gene group in *Δpfap2-p-*40 parasites include protein phosphorylation, actin cytoskeleton organization and fatty acid elongation. Protein phosphorylation has an important role in both egress and invasion in addition to cell cycle control^16,17^. A total of 30 out of 105 genes involved in protein phosphorylation were significantly down-regulated (Extended Data Fig. 4c and Supplementary Data 2), including 10 out of 21 genes encoding FIKK kinases, most of which (17) were exported^18,19^. This suggests a regulatory role for PfAP2-P in signalling processes during egress and invasion (Supplementary Discussion).

### PfAP2-P is essential for trophozoite development

We tested whether gene expression patterns in the bulk RNA-seq data were mirrored at single-cell resolution. Single-cell RNA sequencing (scRNA-seq) was performed using *Δpfap2-p-*16, *Δpfap2-p-*40 and control parasites. Based on the Malaria Cell Atlas (MCA)^20^ annotation, 16 h.p.i. cells are ring/early trophozoite and 40 h.p.i. cells are late trophozoite/schizont parasites (Fig. 2h). We observed that more individual *Δpfap2-p-*16 parasites were classified as ring rather than trophozoite compared with controls (Fig. 2i). However, no difference in stage was observed in *Δpfap2-p-*40 parasites compared with controls, suggesting that RAPA toxicity is not responsible for the observed developmental delay of *Δpfap2-p-*16 parasites. The developmental delay may be an early phenotype resulting from the loss of functional *pfap2-p* expression (Fig. 1o), suggesting that this first peak is critical for trophozoite development. No developmental stage differences between control and *Δpfap2-p*-40 parasites further suggests that the differential expression of genes at 40 h.p.i. (Extended Data Fig. 3b) was due to functional *pfap2-p* deletion and not differences in development or reduced viability.

### The role of PfAP2-P in *var* gene repression confirmed with single cells

In the scRNA-seq data there was significantly higher expression of *var*s (Fig. 2j) and *surfins* (Extended Data Fig. 4d) and down-regulation of *rifins*, *stevors* and *pfmc-2tms* in *Δpfap2-p* parasites, supporting the bulk RNA-seq data (Extended Data Fig. 4d). Therefore, we examined the effect of *pfap2-p* deletion on multiple *var* gene expressions in a single cell. Most control parasites expressed only one *var* gene consistent with the pattern of mutually exclusive expression of *var* genes^15^, but some expressed two or more (Fig. 2k). Significantly more *Δpfap2-p* parasites expressed two or more *var* genes (*P* = 6.23 × 10^−13^, Fisher’s exact test, Fig. 2k), indicating apparent loss of mutually exclusive *var* gene expression.

### *Δpfap2-p* parasites express multiple surface PfEMP1

To determine whether multiple *var* gene expressions in *Δpfap2-p* parasites results in more EMP1s translated and exported to the iRBC surface, we developed a fluorescence-activated cell sorting (FACS)-based assay to detect surface antigens using pooled serum from patients (from 834 Malawian adults) infected with *P. falciparum*^21^. PfEMP1 is a target of naturally acquired antibodies^22^, and therefore if *Δpfap2-p* parasites express multiple PfEMP1s, we expected that they would bind more antibodies in the serum against different PfEMP1s, compared with control parasites. We observed significantly more *Δpfap2-p* iRBCs than control parasites with bound antibody (*P* < 0.0001, two-tailed *t*-test, Fig. 2l, Extended Data Fig. 4e). This result indicates that activation of multiple *var* genes and the translation and transport of PfEMP1 to the iRBC surface occurs in *Δpfap2-p* parasites and suggests that PfAP2-P is involved in silencing *var* gene expression.

### PfAP2-P is a repressor of early gametocyte-associated marker genes

**Extended Data Fig. 5:**
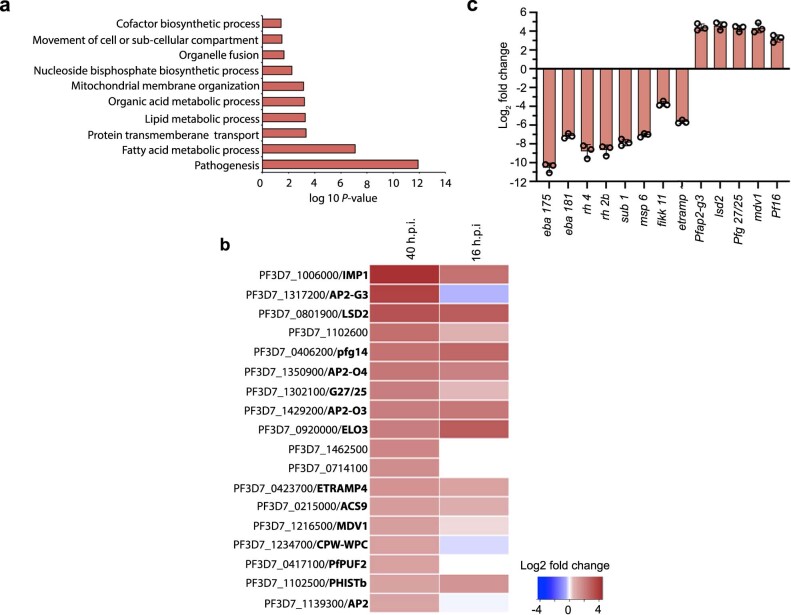
PfAP2-P is a repressor of early gametocyte marker genes. **a**, Gene ontology enrichment analysis of up-regulated genes in *∆pfap2-p* parasites at 40 h.p.i. **b**, Differential expression in *∆pfap2-p* parasites at 40 h.p.i. and 16 h.p.i. of genes that are known or putative early gametocyte markers. **c**, Differential expression of selected genes in RAPA-treated and control parasites (n = 3 biological replicates) measured using qRT-PCR, to validate RNA-seq data; error bar is mean with ± standard error of the mean.

GO analysis of 526 up-regulated genes in *Δpfap2-p*-40 schizonts identified significant enrichment of many biological processes, including lipid and fatty acid metabolism (Extended Data Fig. 5a and Supplementary Data 2). Genes like elongation of fatty acids protein 3 and acyl-CoA synthetase 9 are essential in gametocyte development^13,23^ and were up-regulated in 16 and 40 h.p.i. *Δpfap2-p* parasites (Supplementary Data 1). We examined the list of up-regulated genes for other known or putative early gametocyte marker genes^24,25^ and found 18 out of 28 genes to be up-regulated in both *Δpfap2-p*-16 and 40 parasites (Extended Data Fig. 5b and Supplementary Data 1), suggesting that PfAP2-P is a repressor of commitment to sexual stage development (Supplementary Discussion). We validated the expression of some differentially regulated genes by qRT–PCR (Extended Data Fig. 5c).

### PfAP2-P is a direct regulator of heterochromatin-associated genes

**Fig. 3:**
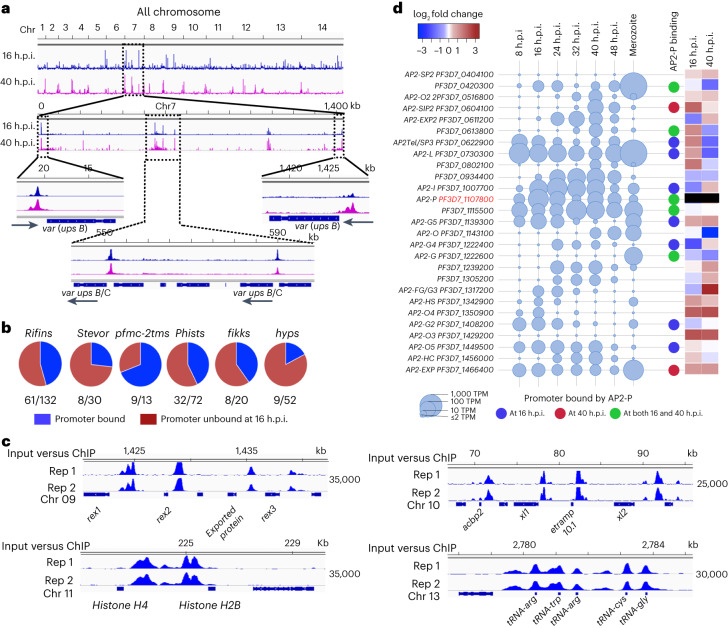
PfAP2-P regulates pathogenesis-associated genes via promoter binding. **a**, Genome-wide occupancy of PfAP2-P at 16 h.p.i. and 40 h.p.i., determined by ChIP–seq. Subtelomeric and internal regions of chromosome 7 (~1,450 kb) containing *var* genes with PfAP2-P bound to their promoter regions are shown as an example. Chromosomal positions are indicated. Results are representative of two independent replicates. **b**, Pie charts showing the proportion of each family of genes with PfAP2-P bound to the promoter region (in blue) at 16 h.p.i. **c**, PfAP2-P occupancy at 16 h.p.i. in putative promoter regions of genes implicated in iRBC remodelling and parasite development. Two biological replicate ChIP versus input tracks are shown (input-subtracted PfAP2-P-ChIP). Positions on chromosome (chr) 09, 10, 11 and 13 are indicated. *x* axis shows the genomic position, and numbers on the right show the enrichment score (Methods). **d**, Expression levels of 27 *P. falciparum* AP2 genes during different IDC stages (AP2-P gene ID highlighted in red) and in merozoites are depicted by the diameter of the circles. On the right, blue, brown and green circles indicate the binding of PfAP2-P to the promoter at either or both 16 h.p.i. and 40 h.p.i.; the heatmap displays the expression status of all 26 *api-ap2s* in ∆*pfap2-p* parasites compared with controls at 16 and 40 h.p.i. The heatmap for *pfap2-p* is black because *∆pfap2-p* parasites only express RNA from the first exon and have no functional AP2-P protein. TPM, transcripts per million.

**Extended Data Fig. 6:**
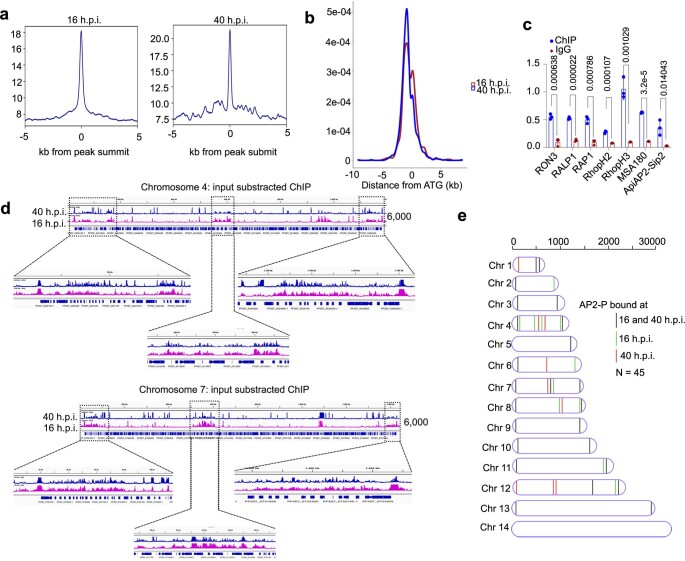
PfAP2-P binds to the putative promoter regions of *var* genes. **a**, Enrichment of PfAP2-P bound reads (from replicate 1) around the ± 5 kb region of peak summits, from 16 h.p.i. (left panel) and 40 h.p.i. (right panel) parasite samples. **b**, The position of ChIP-seq peak summits (common between two biological replicates) relative to the predicted ATG translational start codon, in 16 h.p.i. (red) and 40 h.p.i. (blue) parasites. **c**, Three independent PfAP2-P ChIP experiments followed by qPCR, were performed to validate ChIP-seq data, using selected PfAP2-P-bound promoter regions of genes from samples at 40 h.p.i. The bar-plot shows percent input (% Input) enrichment of PfAP2-P on target genes (mean ± s.d. of three independent experiments). IgG was used as the mock-treated control. *P*-values were calculated using a two-tailed t-test. **d**, Input subtracted ChIP peaks of PfAP2-P in chromosome 4 and 7 as representatives in both 16 and 40 h.p.i. stages. Also, zoomed in PfAP2-P bound central chromosomal and sub-telomeric heterochromatin regions are shown. X-axis shows the genomic position and numbers on the right show the enrichment score. **e**, Schematic diagram of the chromosomal position of *var* genes with promoters bound by PfAP2-P at either 16 h.p.i. (green), 40 h.p.i. (red) or at both stages (black).

To distinguish between direct and indirect targets of PfAP2-P, we performed chromatin immunoprecipitation with the 3HA-epitope tagged PfAP2-P protein followed by DNA sequencing (ChIP–seq). We identified 1,081 and 640 ChIP–seq peaks at 16 and 40 h.p.i., respectively, of which 78% and 68% were in intergenic/promoter regions upstream of at least one gene (with most located less than 2 kb upstream of an ATG translational start site) (Extended Data Fig. 6a,b and Supplementary Data 3). A few PfAP2-P binding regions identified by ChIP–seq at 40 h.p.i. were validated using ChIP–qPCR (Extended Data Fig. 6c). Consistent with a previous finding^26^, binding of PfAP2-P to both central and subtelomeric heterochromatin occurred at both 16 and 40 h.p.i., in addition to binding to the promoter of euchromatic genes (Extended Data Fig. 6d). GO analysis identified encoded proteins enriched in several processes, particularly antigenic variation (*P*_adj_ = 8.36 × 10^−32^ at 16 h.p.i and 6.42 × 10^−38^ at 40 h.p.i.; Supplementary Data 4). PfAP2-P bound significantly (*q* < 0.05) to the promoters of at least 37 and 33 *var* genes at 16 h.p.i. and 40 h.p.i, respectively; a total of 45 *var* genes, including both subtelomeric and internal *var* genes of all the upstream sequence types (Fig. 3a and Extended Data Fig. 6e).

PfAP2-P binding at both 16 and 40 h.p.i. was also enriched at the promoter of genes encoding exportome proteins, with relatively strong signals at 16 h.p.i, and at the promoter or intra-genic regions of many other antigenic variant protein gene families (*rifins*, *stevors*, *surfins* and *pfmc-2tms*) with relatively weaker yet statistically significant signals at 16 h.p.i. (Fig. 3b,c and Supplementary Data 3). Most of these genes were also differentially expressed in *Δpfap2-p* parasites suggesting that they are under direct PfAP2-P control (Supplementary Data 1). These gene families encode for antigenic variant and other exported proteins are located within the central and subtelomeric heterochromatin regions of chromosomes.

The promoters of key genes in early development processes such as translation (*P*_adj_ = 1.3 × 10^−10^), glycolytic process (*P*_adj_ = 0.0005) and nucleosome assembly (*P*_adj_ = 0.02) were also strongly bound by PfAP2-P at 16 h.p.i. (Fig. 3c and Supplementary Data 3 and 4) suggesting PfAP2-P may regulate these processes, which may explain to some extent why *Δpfap2-p*-16 parasites stalled at the late trophozoite/early schizont stage. We compared our data with published ChIP–seq data for PfAP2-P captured at three different stages (that is, ring, trophozoite and schizont)^26^. ChIP–seq peaks identified at 16 h.p.i. show the highest similarity to peaks from ring stages (47% of 16 h.p.i. peaks) with shared genomic regions, and ChIP–seq peaks from the 40 h.p.i. stage had the highest similarity to those from trophozoite stages (41% of 40 h.p.i. peaks) with shared genomic regions (Supplementary Data 5). There is therefore high concordance between our data and published datasets (Supplementary Data 5).

### PfAP2-P binds its own promoter and those of another 13 *apiap2*s

PfAP2-P bound to the promoter of 14 *Pfapiap2* genes, including its own, at both 16 and 40 h.p.i. (Fig. 3d and Supplementary Data 3). Nine of these 13 *apiap2s* were differentially expressed in *Δpfap2-p* parasites at one or both of the timepoints, suggesting that they are under the direct control of PfAP2-P.

**Extended Data Fig. 7:**
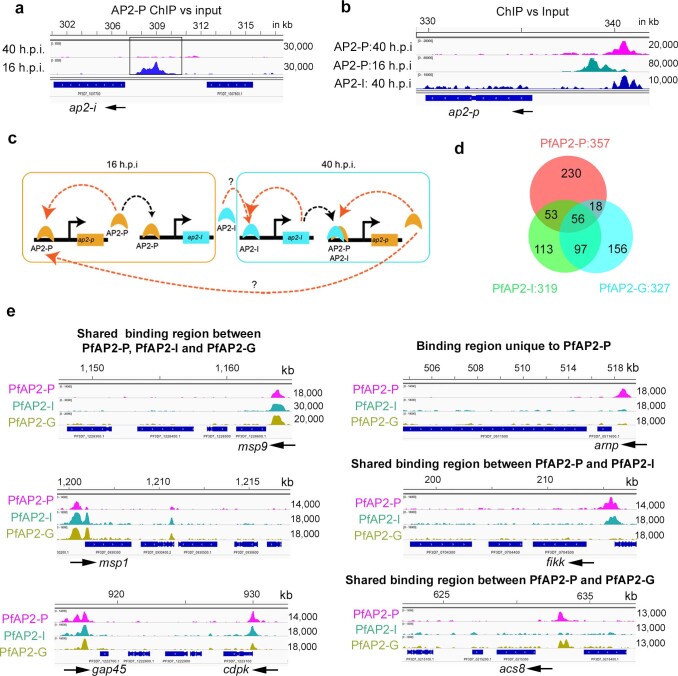
PfAP2-P, PfAP2-I and PfAP2-G binds to many common genomic regions. **a**, Occupancy of PfAP2-P in the promoter region of *pfap2-I* at 16 and 40 h.p.i. **b**, Occupancy of PfAP2-P and PfAP2-I in the promoter region of *pfap2-p* at 16 and 40 h.p.i. **c**, Schematic showing probable gene regulatory network between PfAP2-P and PfAP2-I. Orange arrows indicate the binding of protein to its gene promoter. **d**, Comparison of genes with promoters bound by PfAP2-P (red), PfAP2-I (green) and PfAP2-G (light blue) at 40 h.p.i. **e**, Input-subtracted ChIP-seq read coverage for exemplar genes with promoters that are either bound by all three AP2 proteins (left panel), uniquely by PfAP2-P, by both PfAP2-P and PfAP2-I, or by both PfAP2-P and PfAP2-G (right panel). Arrows show the direction of gene transcription. X-axis shows the genomic position and numbers on the right show the enrichment score.

PfAP2-P bound to the *pfap2-i* promoter (Extended Data Fig. 7a), and *pfap2-i* was down-regulated in *Δpfap2*-16 parasites (Fig. 3d). PfAP2-I binds the *pfap2-p* and its own promoter at 40 h.p.i.^27^, and we observed that PfAP2-P binds to its own promoter at both 16 and 40 h.p.i. (Extended Data Fig. 7b). These data suggest that PfAP2-P expression is autoregulated at both timepoints and that it positively regulates *pfap2-i* expression at 16 h.p.i.; PfAP2-I expression is autoregulated at 40 h.p.i. and it may control *pfap2-p* expression at ~ 40 h.p.i (combinatorial regulation; Extended Data Fig. 7c).

PfAP2-P bound to the same promoter region of many genes as PfAP2-I and PfAP2-G, two AP2s implicated in invasion^27^ and gametocytogenesis^28^, respectively (Extended Data Fig. 7d,e and Supplementary Data 3), suggesting a complex interplay of these AP2s in the control of gene expression (Supplementary Discussion).

### PfAP2-P is an indirect regulator of most invasion- and egress-associated genes

**Extended Data Fig. 8:**
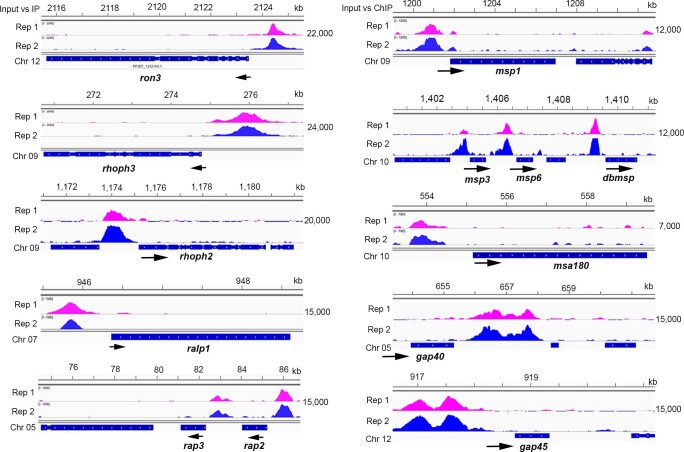
PfAP2-P binds to the putative promoter region of invasion-associated genes. Occupancy of PfAP2-P in the promoter region of different invasion-associated genes. The ChIP tracks show two replicates with input subtracted from the PfAP2-P ChIP data. Arrows indicate direction of transcription. X-axis shows the genomic position and numbers on the right show the enrichment score.

PfAP2-P bound the promoters of few invasion- and egress-associated genes (Extended Data Fig. 8 and Supplementary Data 3) we had identified as down-regulated in *Δpfap2-p-*40 parasites (Supplementary Data 1), suggesting that PfAP2-P directly regulates these genes, but did not bind to the promoters of most (Fig. 2), suggesting that the effects of the *pfap2-p* exon 2 deletion on transcription are both direct and indirect.

### PfAP2-P binds to conserved sequence motifs

**Fig. 4:**
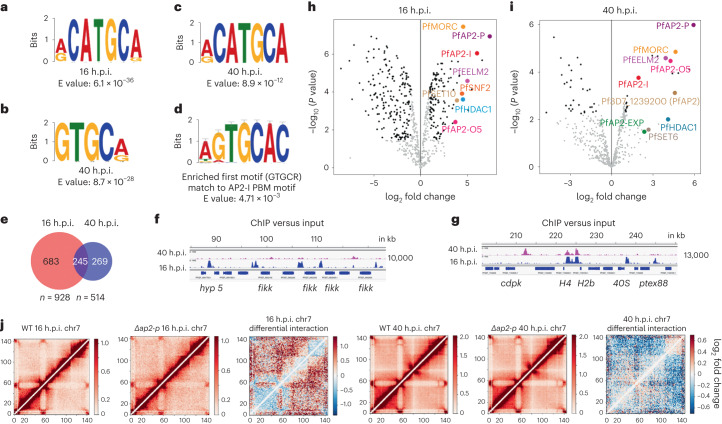
Depletion of PfAP2-P increases chromatin accessibility. **a**, The most significantly enriched motif bound by PfAP2-P at 16 h.p.i. **b**,**c**, The top most (**b**) and the second most (**c**) significantly enriched motifs bound by PfAP2-P at 40 h.p.i. **d**, The most enriched motif (**c**) at 40 h.p.i. is similar to the PfAP2-I binding motif. **e**, The numbers of genes with promoters bound by PfAP2-P at either 16 h.p.i., 40 h.p.i. or both. **f**,**g**, Genomic regions uniquely bound by PfAP2-P at 16 h.p.i. (**f**) or at both 16 and 40 h.p.i. (**g**). *x* axis shows the genomic position and numbers on the right show the enrichment score. **h**,**i**, Label-free quantitative proteomic analysis of *P. falciparum* proteins enriched in PfAP2-P immunoprecipitates at 16 h.p.i. (**h**) and 40 h.p.i. (**i**). **j**, Chromatin contact count heatmap of chromosome 7 at 16 h.p.i. (first three) and 40 h.p.i. (last three) for the control and *Δpfap2-p* parasites, as well as the ${\log }_{2}$ fold change in interactions (third and sixth panels from left) between control and *Δpfap2-p* parasites. Blue indicates a loss of interactions and red indicates an increase of interactions of *Δpfap2-p* over control (WT). Chr, chromosome.

The most significantly enriched sequence motifs bound by PfAP2-P at 16 h.p.i. and 40 h.p.i. were RCATGCR (6.1 × 10^−36^, Fig. 4a), GTGCR (8.7 × 10^−28^, Fig. 4b) and RCATGCA (8.9 × 10^−12^, Fig. 4c), respectively, excluding highly degenerate motifs. GTGCR is significantly similar to the motif bound by PfAP2-I (*P* = 4.7 × 10^−3^, Fig. 4d) and similar to that bound by PfAP2-G (recognizes GTAC as core motif) in vitro^27^, consistent with PfAP2-P binding many sequences bound by PfAP2-I and PfAP2-G (Extended Data Fig. 7d, e). RCATGCR (identified at 16 h.p.i.) is very similar to RCATGCA (identified at 40 h.p.i., *P* = 6.97 × 10^−5^, Fig. 4c), suggesting that PfAP2-P binding is sequence-specific and dynamic with binding to different genomic regions at different IDC stages (Fig. 4e–g and Supplementary Data 3).

### PfAP2-P associates with known and putative epigenetic regulators

To reveal PfAP2-P-associated proteins, we used immunoprecipitation and mass spectrometry (MS) of PfAP2-P-3HA complexes, identifying both known and putative histone modifiers and chromatin remodellers, such as a microrchdia (MORC) family protein, multiple EELM2 domain-containing proteins, imitation protein (ISW1), histone deacetylase 1 (HDAC1), chromodomain-helicase-DNA-binding (CHD1) proteins and five other PfAP2s (Fig. 4h,i and Supplementary Data 6). Many of the proteins were associated with PfAP2-P at both 16 and 40 h.p.i., but some were unique to either timepoint (Supplementary Discussion). The association with PfAP2-I, PfAP2-P and ISWI^27^ is consistent with the observed overlap between PfAP2-P and PfAP2-I binding sites and between the enriched motifs (Extended Data Fig. 7d,e).

### Depletion of PfAP2-P does not alter known histone modifications in heterochromatic regions

**Extended Data Fig. 9:**
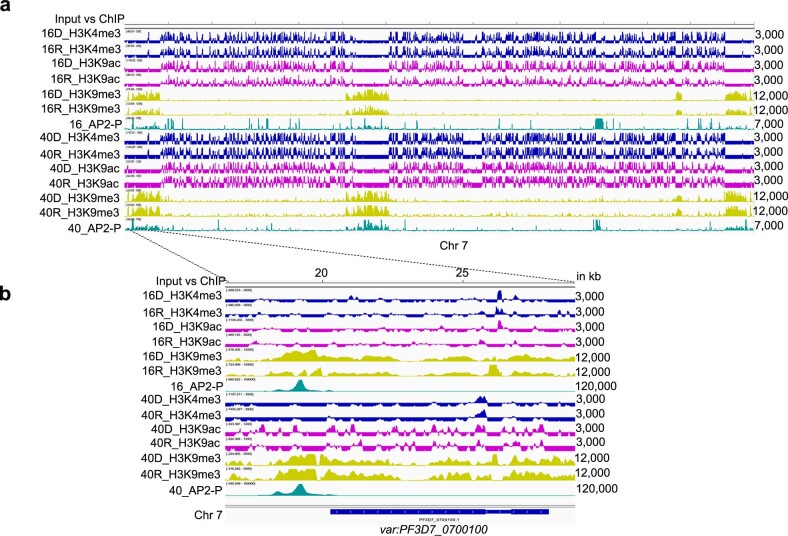
Status of known histone marks in the heterochromatic regions of *pfap2-p* truncated parasites. **a-b**, ChIP-seq profiles of various histone marks (H3K4me3, H3K9ac and H3K9me3) in the chromosome 7 (**a**) and in the promoter and gene body of a *var* gene (**b**) as examples. For ChIP-seq chromatin was sampled at 16 and 40 h.p.i. corresponding to the two peaks of *pfap2-p* expression. We have also provided the binding profile of PfAP2-P at both 16 and 40 h.p.i. for comparison. D;DMSO treated mock control; R: Rapamycin treated *pfap2-p* truncated parasites. Each track is input subtracted ChIP tracks.

Two histone modifications (H3K9ac and H3K4me3) known to be enriched at the active *var* gene promoter^29^ and one modification (H3K9me3) associated with repressed *var* genes^30–32^ were examined. There was clear overlap between H3K9me3 marks and PfAP2-P occupancy in heterochromatic DNA (Extended Data Fig. 9a), but only either slight or no depletion of the H3K9me3 mark in these heterochromatic regions, including within *var* genes, in either *Δpfap2-p*-16 or *Δpfap2-p*-40 parasites, respectively (Extended Data Fig. 9a,b and Supplementary Data 7). No enrichment of the activation marks H3K9ac and H3K4me3 was observed in known heterochromatic regions including in *var* promoters (Extended Data Fig. 9a, b and Supplementary Data 7). These results suggest a new mode of *var* gene regulation with *pfap2-p* and associated chromatin remodellers and histone modifiers independent of the histone marks tested in this study (Supplementary Discussion).

### Depletion of PfAP2-P increases chromatin accessibility

**Extended Data Fig. 10:**
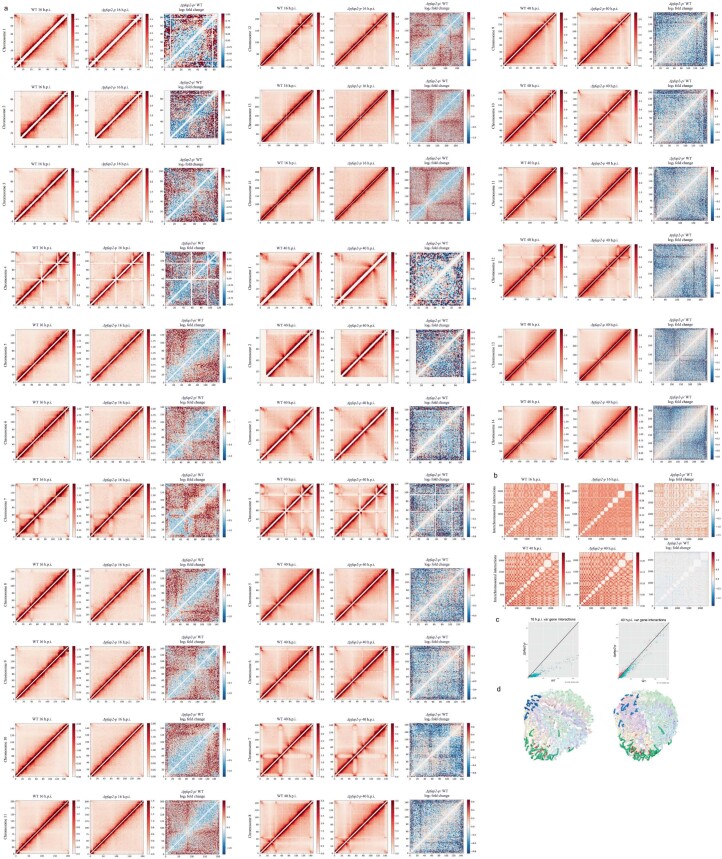
Depletion of PfAP2-P increases chromatin accessibility. **a**, ICE-normalized contact count heatmaps at 10 kb resolution of intrachromosomal interactions for the 14 chromosomes are given for both the 16 h.p.i. and 40 h.p.i. time points. Heatmaps represent a single chromosome of the wild type (left), *Δpfap2-p* (center), and log2 fold change differential interactions (right). The data for the two biological replicates were merged using a weighted average based on the total read count and then counts-per-million normalized prior to generating heatmaps. The scale of the legend was also normalized to improve comparisons between WT and *Δpfap2-p*. For each bin *i*, all interactions within *i* +/−2 are set to 0 (see white line at diagonal) to enhance visualization of remaining bins due to intra-bin and very short-range contacts being significantly higher **b**, Whole-genome interchromosomal contact count heatmaps at 16 h.p.i. (top) and 40 h.p.i. (bottom) for the WT and *Δpfap2-p*. Chromosomes are sorted from left to right and bottom to top. Intrachromosomal interactions are removed. **c**, Number of interactions between *var* gene containing bins. **d**, Whole-genome 3D chromatin models at 16 h.p.i. for the WT (left) and *Δpfap2-p* (right). Centromeres (blue), telomeres (green), and *var* genes (red) are enhanced to display differences between the two samples.

Since disruption of *pfap2-p* has strong effects on genes associated with heterochromatin, we examined the effect of *pfap2-p* inactivation on chromatin structure. We performed a comparative analysis of the chromatin conformation landscape using whole-genome chromosome conformation capture (Hi-C) and deep sequencing from either the 3HA-tagged or the *Δpfap2-p* lines at 16 and 40 h.p.i. Correlation analysis of Hi-C replicates indicated clear reproducibility at both timepoints with overall intra- and inter-chromosomal interaction matrices largely unchanged for each line (Fig. 4j and Extended Data Fig. 10a,b). However, comparative analysis of the tagged and *Δpfap2-p* lines revealed a slight reduction compared with background in long-distance interactions and heterochromatin clusters in *Δpfap2-p*-16 (Fig. 4j and Extended Data Fig. 10a,b). The effect was more global at 40 h.p.i. Genome-wide mapping of interaction changes revealed reduced interaction frequency between telomere ends including internal *var* genes (Extended Data Fig. 10c), consistent with reduced heterochromatin clustering. Genome-wide three-dimensional modelling provided further support for this notion with clear separation of telomeric clustering in *Δpfap2-p* parasites and overall expansion of chromatin structure (Extended Data Fig. 10d). We suggest that chromatin compaction is impaired in PfAP2-P-deficient parasites, increasing chromatin accessibility and increased expression of *var* and genes involved in sexual differentiation. This effect may explain the apparent partial discrepancy between RNA-seq and ChIP–seq data we observe.

Since PfAP2-P binds to the heterochromatic regions and its functional deletion impairs chromatin compaction, we examined whether genes normally only expressed in sporozoites are expressed in *Δpfap2-p* parasites^33^. Of 88 genes apparently expressed specifically in sporozoites^34^, we identified 26 to be significantly up-regulated and 4 to be significantly down-regulated in *Δpfap2-*16 parasites compared with controls (Supplementary Data 1). A similar trend was observed at 40 h.p.i. (26 up- and 15 down-regulated genes). Thus, some sporozoite-specific genes are expressed in *Δpfap2-p* parasites, consistent with PfAP2-P being a general chromatin organizing factor.

## Discussion

**Fig. 5:**
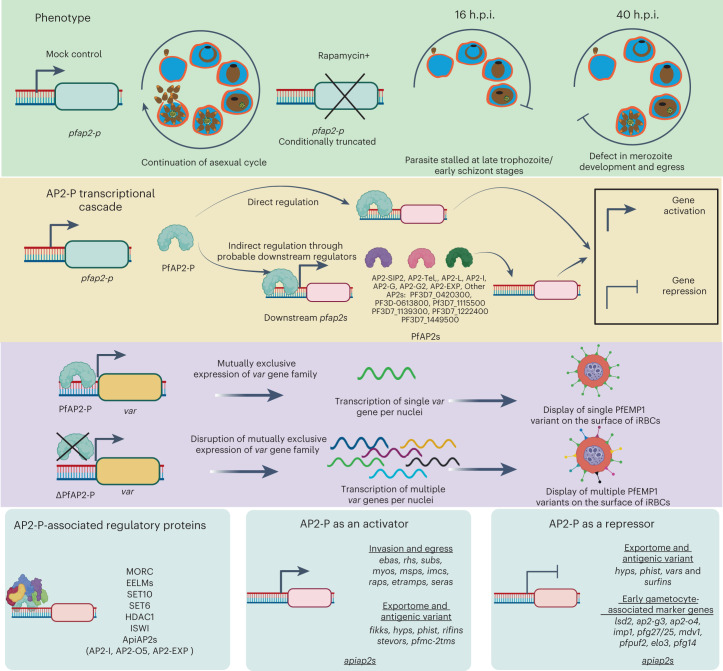
PfAP2-P master regulator of malaria pathogenesis. Deletion of *pfap2-p* before the first peak of expression at 16 h.p.i. blocks parasite development beyond late trophozoite/early schizont stages. Deletion of *pfap2-p* well before 40 h.p.i., but after the first peak of expression at 16 h.p.i., affects merozoite development and blocks parasite egress from iRBCs. At this late stage of intra-erythrocytic development, the second peak of *pfap2-p* expression activates many genes associated with invasion, egress, antigenic variation, host cell remodelling and protein phosphorylation, either directly by binding to their promoter or indirectly through other downstream ApiAP2 transcription factors and regulators. PfAP2-P is a direct repressor of *var* genes and an indirect repressor of many gametocytogenesis-associated marker genes. Deletion of *pfap2-p* derepresses expression of most of *var* genes leading to the display of the corresponding PfEMP1 on the iRBC surface. PfAP2-P acts as a direct activator of many other genes, such as *rifins*, *stevors* and *pfmc-2tms*, coding for antigenically variant proteins, and it binds to the promoter of 14 other ApiAP2s (50% of all *P. falciparum* ApiAP2 genes), suggesting that it is an upstream regulator of gene expression cascades during the IDC. PfAP2-P associates with many known and putative histone modifiers and chromatin remodellers that probably participate in PfAP2-P associated gene regulation. Altogether, PfAP2-P regulates the expression of most known pathogenic factors (associated with antigenic variation and parasite growth) in *P. falciparum*, suggesting it is a master regulator of malaria pathogenesis.

We provide multiple lines of evidence that PfAP2-P is an essential regulator of key processes in malaria parasite development and pathogenesis during the IDC (Fig. 5). Functional deletion of *pfap2-p* just before each of its two expression peaks showed it has distinct essential roles at each timepoint. The first peak at 16 h.p.i. is critical for parasite development beyond trophozoites, while the second peak at 40 h.p.i. is indispensable for merozoite development and egress (Fig. 5 and Supplementary Discussion). PfAP2-P represses *var* gene expression at both timepoints, either by interacting with their promoter or by regulating chromosomal accessibility through maintenance of chromatin compaction (Supplementary Discussion). In single *Δpfap2-p* parasites, activation of most silenced *var* genes was detected at 16 h.p.i. Increased *P. falciparum*-specific antibody recognition of proteins on the surface of iRBCs containing *Δpfap2-p* parasites suggested that there was more surface PfEMP1 from translated transcripts of multiple *var* genes. Together, these results suggest that PfAP2-P has a crucial role in silencing most *var* genes and maintaining the mutually exclusive expression pattern of this gene family.

This study establishes that a key gene regulator, PfAP2-P, controls several important processes in malaria parasite development and pathogenicity, paving the way to further understand the mechanisms regulating gene expression. It suggests novel therapeutic strategies, such as using *Δpfap2-p* parasites as a live anti-disease vaccine, expressing the PfEMP1 repertoire to elicit immune responses to diminish malaria-associated pathology.

## Methods

### Parasite culture, maintenance, synchronization and transfection

The DiCre-expressing *P. falciparum* clone II3 (ref. ^10^) was maintained in human A+ erythrocytes at 37 °C in Roswell Park Memorial Institute 1640 medium containing AlbumaxII (Invitrogen) supplemented with 2 mM l-glutamine. Parasites were either synchronized by sorbitol treatment or by purifying mature schizont stages using 70% (v/v) isotonic Percoll (GE Healthcare Life Sciences) before allowing re-invasion to occur, followed by sorbitol treatment. For transfection of plasmid constructs, purified Percoll-enriched mature schizonts (~20 μl packed volume) were suspended in 100 μl of P3 primary cell solution (Lonza), containing 60 μg of linearized repair plasmid DNA (repair plasmid 1 and 2 separately in two separate transfection experiments) and 20 μg of pDC2 plasmid with the required cloned guide DNA and electroporated using an Amaxa 4D-Nucleofactor (Lonza) using programme FP158 as previously described^35^. Drug selection was applied ~20 h after transfection with 5 nM WR99210 (a kind gift of Jacobus Pharmaceuticals) for 4 days. Once sustained growth of drug-resistant transgenic parasites was observed, the cultures were treated with 1 μM 5-fluorocytosine provided as clinical Ancotil (MEDA) for 4 days. Transgenic parasite clones PfAP2-P:loxP and PfAP2-P-3HA:loxP were obtained by limiting dilution cloning in microplates. DiCre recombinase-mediated excision was induced by adding rapamycin to the culture at a final concentration of 10 nM.

### Strategy to perform conditional exon 2 deletion (gene truncation) of PfAP2-P

To excise exon 2 of *pfap2-p*, which contains the DNA-binding AP2 domain and nuclear localization signal, the endogenous exon 2 of *pfap2-p* in the II3 DiCre-expressing *P. falciparum* clone was replaced with transgenic, ‘floxed’ and HA-tagged and non-tagged forms of the exon 2 using two sequential Cas9-mediated genome editing procedures. In the first step, the single intron of *pfap2-p* was replaced with *sera2*-loxPint^36^. The repair plasmid (repair plasmid 1) was synthesized commercially (GeneArt; Life Technologies) with re-codonized sequences in the 2,986–3,024 bp and 3,273–3,305 bp regions of the *pfap2-p* and 400 bp homology arms flanking the 5′ and 3′ regions of the intron. In the second step, cloned parasites with the integrated *sera2-loxPint* were used to introduce a further loxP immediately after the stop codon of the *pfap2-p* gene. The repair plasmid for this (repair plasmid 2) contained re-codonized sequences in the gene’s 6,261–6,330 bp region, a *XmaI* restriction enzyme site followed by a loxP sequence just after the TAA stop codon, and 400 bp homology regions.

A triple HA-tagged version of the PfAP2-P was also prepared. For this, the stop codon was removed from the donor sequence (repair plasmid 2) by PCR amplification using primer pairs (oligos P1and P2) and digested with *NotI* and *XmaI* restriction enzymes. To remove the sequence with the stop codon from the plasmid backbone, repair plasmid 2 was also digested with *Not1* and *XmaI* restriction enzymes. Digested, amplified sequence without stop codon and digested plasmid backbone were ligated to get the repair plasmid 2 without stop codon after the PfAP2-P coding sequence. A triple HA tag sequence was PCR amplified from pFCSS plasmid with *XmaI* sites on both the sides of the HA tag sequence using oligos P5 and P6. *XmaI-*digested repair plasmid 2 without stop codon was ligated with *XmaI-*digested triple HA tag fragment to generate a triple HA-tagged encoding repair plasmid 2.

pDC2–Cas9–U6–hdhfr plasmid expressing pyogenes Cas9 was used in this study. Guide RNA sequences were identified using Benchling. The TATTTATATTCTCAATTGAA and TTATATTCTCAATTGAATGG sequences targeting the 3′ end of the first exon, upstream of the TGG protospacer-adjacent motif and 5′ end of the second exon upstream of the TGG protospacer, respectively, were cloned into the pDC2-Cas9-U6-hdhfr (pDC2 plasmid 1) and used to target Cas9 in the first Cas9-mediated editing step (Fig. 1a). The CCCTTCAATAGATTCGCACA sequence towards the 3′ end of the second exon, upstream of the CGG protospacer-adjacent motif, was cloned into the pDC2-Cas9-U6-hdhfr (pDC2 plasmid 1) and used to target Cas9 in the second Cas9-mediated editing step. Sequences of all the oligonucleotides and primers used are listed in Supplementary Data 8.

### Flow cytometry-based quantification of parasitaemia

Parasites were fixed in 0.1% glutaraldehyde, incubated for 30 min at 37 °C, then stored at 4 °C until further use. Glutaraldehyde fixed parasites were stained with SYBR Green (diluted 1:10,000) for 30 min at 37 °C, then parasitaemia was determined using a BD LSR Fortessa flow cytometer (BD Biosciences).

### Invasion and growth assays

For the invasion assay, the parasites were treated with DMSO or rapamycin at ~16 h.p.i. in cycle 0 and mature schizonts in the same cycle were isolated using Percoll as described earlier and added to the culture at 5% parasitaemia. Merozoites were allowed to invade for 4 h under either static or vigorous shaking (250 rpm) conditions. A total of 4 h after adding mature schizonts to the culture, the parasitaemia was measured by flow cytometry. Three biological replicates per condition were used.

### Electron Microscopy

Compound 2-treated and RAPA-treated (parasites with disrupted second peak of *pfap2-p* expression) highly synchronized parasites were allowed to grow until they reached the segmented schizont stage. Then the iRBCs were fixed with 2.5% glutaraldehyde in cacodylate buffer (0.1 M, pH 7.4) for 48 h. A first osmication was performed using reduced osmium (1:1 mixture of 2% osmium tetroxide and 3% potassium ferrocyanide) for 1 h. After a quick wash with distilled water, a second osmication was performed with 2% osmium tetroxide for 30 min. Samples were washed 3 × 5 min in water and placed in 1% uranyl acetate for 12 h at 4 °C. Before dehydration, samples were washed 3 × 15 min in water. After pre-embedding in 1% agar, samples were dehydrated in an ethanol series (40% to 100%) and embedded in epoxy resin. Thin sections (100–50 nm thick) were collected on copper grids and contrasted with lead citrate. Imaging was performed using a transmission electron microscope operating at 300 kV (Titan Cryo Twin, Thermo Fisher Scientific). Images were recorded on a 4k × 4k charged-coupled device camera (Gatan Inc.). In 50 randomly selected iRBCs from each group, the parasites were categorized as either mature schizont, segmenter or dead.

### Nucleic acid extraction and polymerase chain reaction

For DNA isolation, cells were pelleted and treated with 0.15% saponin in phosphate-buffered saline (PBS) for 10 min on ice, then washed twice with PBS. DNA was extracted from parasite pellets using DNeasy blood and tissue kit (Qiagen) following the manufacturer’s instructions. For diagnostic PCR to check clones, GoTaq (Promega) DNA master mix was used, for amplification of fragments (3HA tag) used in construct design, Phusion high fidelity DNA polymerase (NEB) was used, and for amplification of fragments longer than >3 kb Platinum taq HiFi polymerase (Invitrogen) was used.

### PAGE, immunoblotting and immunofluorescence

Ring-stage parasites were treated with DMSO or rapamycin, and subsequently, mature schizonts (>45 h.p.i) were purified using 70% Percoll, then treated with 0.15% saponin in PBS and washed twice with PBS. Schizonts pellets were lysed by adding sample lysis buffer (1% NP-40, 0.1% sodium dodecyl sulfate (SDS) and 150 mM NaCl), and 5 µg protein of each sample were separated under reducing conditions on Bis-Tris NuPAGE polyacrylamide gels and transferred to nitrocellulose membranes by electroblotting. Blots were blocked overnight in 5% milk powder (w/v) in PBS containing 0.2% Tween-20. To detect 3HA-tagged PfAP2-P, the rat anti-HA mAb 3F10 (Sigma) was used at a 1:1,000 dilution, followed by horseradish-conjugated secondary antibody (1:2,500). For the proteins tested, relevant primary antibodies were used (see below), then secondary horseradish peroxidase-conjugated antibodies specific for mouse, rabbit or rat IgG (Bio-Rad) were used at a dilution of 1:2,500. The signal was developed using Immobilon Western Chemoluminescent HRP Substrate (Merck Millipore) and detected using Hyperfilm ECL film (GE Healthcare).

For immunofluorescence, thin parasite films were prepared and fixed in 4% paraformaldehyde in PBS for 20 min at room temperature (25 °C). The cells were permeabilized with 0.1% (v/v) Triton ×100 in PBS for 5 min, then blocked with 3% bovine serum albumin (BSA) in PBS overnight at 4 °C before being probed with relevant primary antibodies. Secondary Alexa Fluor 488- or 594-conjugated antibodies specific for mouse, rabbit or rat IgG (Invitrogen) were used at a 1:5,000 dilution. Slides were examined using a Nikon Ni microscope with a 100× Plan Apo NA 1.45 objective; images were captured with an Orca Flash 4 digital camera and prepared with Nikon NIS Elements and Adobe Photoshop software (2021).

### Antibodies

The following antibodies and dilutions were used for western blots in these studies: rabbit anti-EBA175 (1:10,000), rat anti-MyoA (1:1,000), rat anti-binding immunoglobulin protein (BiP) (1:1,000), rabbit anti-PTRAMP (1:4,000), rabbit anti-ARO (1:1,000), rabbit anti-AMA1 (1:10,000) and rabbit anti-SUB1 (1:1,000). Antibodies used in immunofluorescence were rabbit anti-GAP45 (1:1,000) and rabbit anti-MSP7 (1:1,000). Anti-EBA175 was obtained from MR4 BEI Resources, anti-BiP was provided by Dr E. Knuepfer, anti-SUB1 was a generous gift from Prof. M. Blackman (Francis Crick Institute), and anti-AMA1 was a generous gift from B. Faber and C. Kocken from the Primate Research Centre in Rijswijk. All other antibodies were generated in the Holder laboratory and are now held and freely available at NIBSC-CFAR (please contact cfar@nibsc.org with any inquiries).

### RNA extraction and strand-specific RNA-seq library preparation

Parasite cultures (~0.5–2 ml, depending on the asexual developmental stage) were pelleted at 2,400 rpm for 3 min, lysed by adding 1 ml of TRIzol (Sigma), then immediately stored at −80 °C until further use. Total RNA was isolated from TRIzol-lysed parasites following the manufacturer’s instructions (Life Technologies). Strand-specific messenger RNA libraries were prepared from total RNA using TruSeq Stranded mRNA Sample Prep Kit LS (Illumina) according to the manufacturer’s instructions. Briefly, for each sample, 100–300 ng of total RNA was used to prepare the libraries. PolyA+ mRNA was captured from total RNA using oligo-T attached to magnetic beads. First-strand synthesis was performed using random primers followed by second-strand synthesis where dUTP was incorporated in place of dTTP to achieve strand-specificity. Double-stranded complementary DNA ends were ligated with adaptors, and the libraries were amplified by PCR for 15 cycles before sequencing the libraries on the Illumina HiSeq-4000 platform with paired-end 150 bp read chemistry according to manufacturer’s instructions (Illumina).

### RNA-seq data processing and analysis

The quality of the raw reads was assessed using FASTQC v0.11.8 (ref. ^37^). Low-quality reads and Illumina adaptor sequences from the read ends were removed using TrimmomaticR (ref. ^38^). Processed reads were mapped to the *P. falciparum* 3D7 reference genome (release 40 in PlasmoDB, http://www.plasmodb.org) using Hisat2 (ref. ^39^) (v2.1.0) with parameter ‘—rna-strandness FR’. Counts per feature were estimated using FeatureCounts^40^. Raw read counts data were converted to counts per million, and genes were excluded if they failed to achieve a counts per million value of 1 in at least one of the three replicates performed. Library sizes were scale normalized by the trimmed mean of *M*-values method using EdgeR software^41^ and further subjected to linear model analysis using the voom function in the limma package^42^. Differential expression analysis was performed using DeSeq2 v1.38.3 (ref. ^43^). Genes with an FDR-corrected *P* value (Benjamini–Hochberg procedure) <0.05 and log_2_ fold change ≥1 or ≤−1 were considered as up-regulated or down-regulated, respectively.

### Real-time quantitative PCR

Total RNA was treated with DNAase (TURBO DNase, cat. no. AM2238, Invitrogen) following the manufacturer’s instructions. The removal of DNA was confirmed by performing PCR using housekeeping genes. cDNA (1 µg) was prepared from total RNA using LunaScript cDNA synthesis mix (NEB) and diluted five times before using it for qRT–PCR. mRNA expression levels were estimated on a Quant Studio 3 qRT–PCR machine (Applied Biosystems) using Fast SYBR Green master mix (Applied Biosystems, cat. no. 4385612). Seryl-tRNA ligase (PF3D7_0717700) was used as the internal control to normalize mRNA levels. Specific amplification of the PCR product was verified by dissociation curve analysis and relative quantities of mRNA calculated using the ∆∆Ct method^44^. PCR primers used in the qRT–PCR experiment are listed in Supplementary Data 8. For the *var* gene qRT–PCR experiment, primer sets targeting individual *var* genes described elsewhere^45^ were used, including the primers targeting the seryl-tRNA ligase gene (PF3D7_0717700) used as the internal control.

### PfAP2-P ChIP (AP2-P ChIP)

The ChIP assay was performed as described^46^ with a few modifications. Parasite culture (50 ml) containing synchronized ∼16 h.p.i. or ~40 h.p.i. parasites at ~5% parasitaemia was centrifuged at 900*g* for 4 min, and the cells were washed once with PBS. A total of 25 ml of 0.15% saponin in PBS was added to the cell pellet and incubated on ice for 10 min, followed by washing it twice with cold PBS. Parasites were cross-linked for 10 min by adding methanol-free formaldehyde at 1% final concentration and incubated for 10 min at 37 °C with occasional shaking. The cross-linking reaction was quenched by adding 1.25 M glycine to a final concentration of 0.125 M and incubated at 37 °C for another 5 min. Parasites were centrifuged for 10 min at 3,250*g* at 4 °C, washed three times with DPBS, snap-frozen in liquid nitrogen, and stored at −80 °C until further use. Frozen formaldehyde-fixed parasites were thawed on ice for ChIP. A total of 1 ml of nuclear extraction buffer (10 mM *N*-2-hydroxyethylpiperazine-*N*′-2-ethanesulfonic acid, 10 mM KCl, 0.1 mM ethylenediaminetetraacetic acid (EDTA), 0.1 mM ethylene glycol tetraacetic acid, 1 mM dithiothreitol and 1× EDTA-free protease inhibitor cocktail (Roche)) was added to the tubes containing thawed parasites and incubated on ice for 30 min. After the incubation, 10% NP-40 was added to reach a final concentration of 0.25%, and the parasites were lysed by passing through a 26^1/2^ G needle seven times. Parasite nuclei were collected by centrifuging at 2,500*g* for 10 min at 4 °C. Shearing of chromatin was carried out using the Covaris Ultra Sonicator (E220) for 14 min with the following settings: 5% duty cycle, 140 intensity peak incident power, and 200 cycles per burst to obtain fragment size of 200–600 bp. Insoluble materials were removed by centrifuging the sheared chromatin for 10 min at 13,500*g* at 4 °C. A total of 30 µl of fragmented chromatin were stored as input at −80 °C.

Fragmented chromatin was diluted 1:1 in ChIP dilution buffer (30 mM Tris–HCl pH 8.0, 0.1% SDS, 3 mM EDTA, 300 mM NaCl, 1% Triton X-100, and EDTA-free protease inhibitor cocktail). Chromatin was incubated overnight with 6 µg rabbit polyclonal anti-HA (Abcam no. ab9110) or, for the control, the same amount of rabbit IgG isotype control (cat. no. 10500c, Invitrogen). For histone marks H3K9me3, H3K9ac and H3K4me3, rabbit polyclonal anti-H3K9me3 antibody (Millipore no. 07–442), rabbit polyclonal anti-H3K9ac antibody (Millipore no. 07–352) and rabbit polyclonal anti H3K4me3 antibody (Abcam no. ab8580) were used, respectively. The antibody–protein complex was recovered with protein A coupled to magnetic beads (Dynabeads, Invitrogen, cat. no. 10002D), followed by extensive washes with low salt immune complex wash buffer, high salt immune complex wash buffer (washes done at 4 °C), and Tris and EDTA buffer (washes done at room temperature). Chromatin was eluted with elution buffer (1% SDS, 0.1 M NaHCO_3_) at 45 °C for 30 min with shaking. Immunoprecipitated chromatin and input were reverse cross-linked overnight at 45 °C by adding 5 M NaCl to a final concentration of 0.5 M. Samples were treated with RNase A for 30 min at 37 °C, followed by a 2 h incubation at 45 °C with proteinase K (final concentration 66 µg ml^−1^). DNA was purified using ChIP DNA Clean and Concentrator (Zymo Research, cat. no. D5205).

### PfAP2-P ChIP–seq and analysis

Libraries were prepared using NEBNext Ultra II DNA library kit following the manufacturer’s instructions until the step of adapter ligation (adapters were diluted at 1:20 ratio). Adapter-ligated libraries were purified using AmpureXP beads. The libraries were amplified for a total of six PCR cycles (2 min at 98 °C initial denaturation; six cycles of 30 s at 98 °C, 50 s at 62 °C, and final extension 5 min at 62 °C) using the KAPA HiFi HotStart Ready Mix (Kapa Biosystems). Amplified libraries were purified, size selected for 350 bp inserts using AmpureXP beads, and sequenced on the Illumina HiSeqX platform with 150 bp paired-end read layouts. Low-quality reads and Illumina adaptor sequences from the read ends were removed using Trimmomatic v0.33 (ref. ^38^). Quality-trimmed reads were aligned to the *P. falciparum* genome (http://www.plasmodb.org, v3, release v32) using Hisat2 v2.1.0. Duplicate reads were removed using samtools v1.8 (markdup)^47^. GC bias was corrected using deeptool’s correctGCBias tool^48^. For coverage plots of AP2-P 40 h.p.i. and 16 h.p.i. ChIP–seq experiments, deeptool’s bamCompare tool was used to normalize the read coverage per base of the genome position (option ‘-bs 1’) in the respective input and ChIP samples or IgG and ChIP samples to the total number of reads in each library (–normalizeUsing RPKM). Normalized input coverage or IgG coverage per bin was subtracted from the ChIP values (option–operation subtract). Coverage plots were visualized using IGV genome browser v2.4.16 (ref. ^49^).

ChIP peaks (*q* value cut-off <0.05) were identified using macs2 v1.4.2 (ref. ^50^) by comparing the input with ChIP or IgG with ChIP with default settings but without prior peak modelling (option ‘-nomodel’), the fragment size set to 200 bp (option ‘-extsize 200’) and the genome size (option ‘-g’) set to 233332839. Robust common peaks between replicates were identified using bedtools v2.29.0 ‘intersect’ (option –f 0.30 –r) (ref. ^51^). Peak annotation was carried out using Homer’s annotatePeaks.pl that assigned each peak with the nearest downstream gene. After intersecting, common peaks with peak score >50 were kept for further analysis. Enrichment heatmaps and profile plots were generated using the deepTools v2.29.0 computeMatrix and plotHeatmap tools.

### Processing of published PfAP2-I and PfAP2-G ChIP–seq data

PfAP2-I and PfAP2-G ChIP–seq published raw data^27,28^ were downloaded from European Nucleotide Archive (ENA) and processed exactly as the ChIP–seq data for PfAP2-P. ChIP peaks (*q* value cut-off <0.05) were identified using macs2 v1.4.2 (ref. ^50^) by comparing the input with ChIP for both the replicates of PfAP2-G and PfAP2-I. Robust common peaks between PfAP2-P and PfAP2-I, PfAP2-P and PfAP2-P, and between all three were identified using bedtools v2.29.0 ‘intersect’ (option –f 0.30 –r) to find peaks that overlapped at least 30%.

### Processing of published PfAP2-P (PfAP2-11A) ChIP–seq data

Published PfAP2-P (PfAP2-11A) ChIP–seq raw data^26^ were downloaded from ENA and processed exactly as the ChIP–seq data for PfAP2-P. ChIP peaks (*q* value cut-off <0.05) were identified using macs2 v1.4.2 (ref. ^50^) by comparing the input with ChIP for both the replicates of PfAP2-P (published)^26^. Robust common peaks of PfAP2-P from this and the published study^26^ were identified using bedtools v2.29.0 ‘intersect’ (option –f 0.30 –r) to find peaks that overlapped at least 30%. Peak annotation was carried out using Homer’s annotatePeaks.pl (3.2.1) that assigned each peak with the nearest downstream gene. After intersecting, common peaks with peak score >50 were kept for further analysis.

### Parasite sample preparation for scRNA-seq

For the 40 h.p.i. timepoint, tightly synchronous parasites were enriched using 63% Percoll, washed twice with incomplete Roswell Park Memorial Institute 1640 medium, and processed immediately on the 10X Chromium controller (10X Genomics). For the 16 h.p.i. timepoint, parasites were stained with Mitotracker Deep Red FM (Life Technologies, #M22426) for FACS analysis and flow sorting, respectively. Briefly, 50 µl of SYBR Green I stained RBCs were analysed on BD LSR Fortessa Flow Cytometer with High Throughput sampler (BD Biosciences) using BD FACS Diva Software v6.2 and 488 laser excitation/530 emission filter to determine the concentration of SYBR Green I positive cells µl^−1^. A BD Influx Cell Sorter (BD Biosciences) with BD FACS Sortware v1.0.01 software was used to sort ~40,000 MitoTracker Deep Red FM-positive RBCs using a 70 µm nozzle, a 640 nm laser excitation/670 nm emission filter, and a pressure setting of 30 psi. Post-sorted cell concentration and quality were checked using a Countess II Automated Cell Counter (Invitrogen) and FLoid Cell Imaging Station (Thermo Fisher Scientific). Finally, labelled cells (that is, SYBR Green I or MitoTracker Deep Red FM-positive cells) were then loaded onto a 10X chip (Chip G) and processed immediately on the 10X Chromium controller (10X Genomics).

### scRNA-seq library preparation

Single-cell libraries were constructed using the 10X Genomics Chromium Next GEM Single Cell 3′ Reagent Kits v3.1 with Single Index Kit T Set A. Due to the extremely low RNA content of the single-cell malaria parasite and the AT-rich genome (~70% AT), modifications to the cDNA amplification and library preparation workflow were made accordingly. These modifications included: 30× cDNA amplification cycles, taking 50% cDNA as input into library generation, reducing fragmentation time to 2 min, and changing the extension time to 65 °C during index PCR. Individual library quality control was performed using the BioAnalyzer HS DNA Assay kit (Agilent).

### Sequencing of scRNA-seq library

Library concentration was determined with the KAPA Library Quantification Kit (Roche) using the QuantStudio3 Real-Time PCR systems (Thermo Fisher Scientific) and assessed for fragment size using the BioAnalyzer HS DNA Assay kit (Agilent). Following library pooling in equimolar concentrations, a total of 1.2 nM library was sequenced on the Illumina NovaSeq 6000 with SP flow cell using version 1.5 chemistry as follows: read 1–28 bp, index read i7–8 bp, index read i5–0 bp, and read 2–91 bp.

### Single-cell transcriptome alignment and read count estimation

The droplet-based sequencing reads were aligned to the hybrid human genome hg38 and *P. falciparum* pd37 (PlasmoDB-46_Pfalciparum3D7_Genome.fasta) to remove any human transcript contamination. This was achieved using CellRanger v5.0.1 standard pipeline using --nosecondary flag. The raw gene count matrix was subjected to various single-cell pre-processing steps separately.

### Pre-processing and normalization

Primarily, the reads mapping to human genes were removed, followed by the identification and removal of empty droplets using the emptyDrops() function from R package dropletUtils v1.12.3 (ref. ^52^). This function determines whether the RNA content of a cell barcode differs considerably from the ambient background RNA present in each sample. Cells with FDR ≤ 0.001 (Benjamini–Hochberg-corrected) were examined for subsequent investigation. The per cell quality metrics were computed by the addPerCellQC function of the scuttle package v1.2.1 (ref. ^53^). The deconvolution approach in the computeSumFactors function of the Scran R package v1.20.1 (ref. ^54^) was utilized to normalize cell-specific biases. We kept the mitochondrial genes of *P. falciparum* since the proportion of unique molecular identifiers allocated to mitochondrial genes in both control and conditional truncated lines were similar.

Further, the doublets identification was performed using the computeDoubletDensity function of the scDblFinder Bioconductor package v1.6.0 (ref. ^55^) (scDblFinder: scDblFinder R package version 1.6.0 (ref. ^56^)). This was achieved in three steps. (1) The log normalization of counts was achieved using the logNormCounts function of scuttle package. (2) The modelGeneVarByPoisson function of scran^54^ was then used to model the per gene variance, followed by (3) doublet score calculation using the top 10% of highly variable genes. We then cleaned our data based on a 95% quantile cut-off. Additionally, the standard functions of the Seurat package v4.1.1 (ref. ^57^) were also used to generate intuitive quality control plots.

### Cell type and infection stage recognition

The transcriptomic data at single-cell resolution of the malaria life cycle was obtained from the MCA. The phenotypic data for MCA were obtained from refs. ^58,59^. We used the SingleR package v1.6.1 to transfer labels from the reference atlas to each cell in our data. It identifies marker genes for each stage in the reference atlas and uses them to compute assignment scores (based on the Spearman correlation across markers) for each cell in the test dataset against each label in the reference. The top 20 marker genes were identified using the Wilcoxon rank sum test^60^.

The time-series data of the IDC was obtained from Subudhi, A.K. et al.^11^. To transfer the time-series labels from Bulk RNASeq, we used a subset of samples for both 16 h.p.i and 40 h.p.i to prevent label misassignments. Each biological replicate was considered a single cell and markers were identified using the ‘classic’ method of SingleR v1.20.1.

The correlation between cells of different stages was carried out using the CorrelateReference function of the CHETAH R package v1.8.0 (ref. ^61^). Similarly, we performed an inter-sample correlation between the transcriptional profiles of samples from different timepoints.

### Integration of in-house data with MCA

The cleaned data were then integrated with MCA single cell data using standard scRNA-seq integration workflow as described elsewhere^57^. Briefly, we create an ‘integrated’ data assay, but first identify the pairwise anchors using FindIntegrationAnchors followed by IntegrateData that exploit this anchor set to combine the MCA and in-house data. Next, we ran the standard workflow on the integrated assay, including scaling, dimension reduction and clustering, using parameters described in ref. ^62^. The clusters were then manually annotated using the RenameIdents function of Seurat^57^.

### *var* gene expression per cell

A set of 61 *var* genes were used for their average expression calculation. The *var* gene expression was calculated using raw counts (RNA assay data slot). *var* gene expression calculation equals the sum of *var* gene expression divided by the sum of expression of all genes.

### PfAP2-P motif identification

Sequences from the commonly identified peaks from two replicates were extracted from the *P. falciparum* 3D7 genome using the bedtools v2.29.0 ‘getfasta’. These retrieved sequences were then uploaded to the DREME server^63^ to identify significantly enriched motifs in the peak region. Tomtom^64^ was used to compare the de novo identified motifs to previously in silico discovered motifs^65^.

### Immunoprecipitation of PfAP2-P and identification of proteins by MS

To identify the AP2 interacting proteins, the on-beads digestion with trypsin approach was used after immunoprecipitation of AP2 complex. Before performing the on-beads digestion, after the Tris and EDTA buffer washes, the immunoprecipitated complex was washed twice with exchange buffer (100 mM NaCl and Tris 50 mM pH 7.5) for 10 min at 4 °C. Subsequently, the beads were resuspended in 100 μl of 100 mM triethylammonium bicarbonate, and a reduction of bound proteins was done with 1 mM dithiothreitol at 37 °C for 30 min on thermo mixture with constant shaking at 750 rpm. The sample was brought to room temperature and alkylated in the dark with 3 mM iodoacetamide for 45 min; the excess iodoacetamide was quenched with 3 mM 1,4-dithiothreitol (DDT) for 10 min (Sigma Aldrich). Afterwards, the sample was digested with 2.5 μg trypsin (Promega) overnight on a thermo mixture with constant shaking at 1,000 rpm. The resulting digested peptides were purified from beads, and trypsin digestion was stopped by adding trifluoroacetic acid (TFA) to a 2% final concentration. The acidified peptide was desalted using Sep-Pak C18 1 cc Vac Cartridge, 50 mg Sorbent per Cartridge (Waters). Briefly, the Sep-Pak column was conditioned with 1 ml 100% methanol twice and equilibrated with 1 ml of 0.1% TFA twice. After that, the digested acidified peptides were loaded. The bound peptides were washed with 1 ml of 0.1% TFA twice and eluted with 300 μl of elution buffer (acetonitrile 75% with 0.1% TFA in water) twice. The eluted peptides were dried in SpeedVac and kept at −80 °C until further use.

### LC–MS analysis of peptides

The liquid chromatography (LC)–MS analysis was performed on Q-Exactive HF mass spectrometer coupled with an UltiMate 3000 UHPLC (Thermo Fisher Scientific). The peptides were dissolved in 0.1% formic acid (FA; Sigma Aldrich), and approximately 1 μg of peptides was separated on an Acclaim PepMap C18 column (75 μm isotope dilution × 250 mm, 2 μm particle sizes, 100 Å pore sizes) with a gradient of 5–35% mobile phase A and B, respectively, for 55 min, ramping up to 90% phase B for 5 min, and the column was conditioned to 2% phase B for 10 min with the flow rate of 300 nl min^−1^ (phase A is 0.1% FA and phase B is 99.9% acetonitrile with 0.1% FA). The peptides were introduced into the mass spectrometer through Nanospray Flex with an electrospray potential of 2.5 kV. Data were acquired in the Orbitrap at the resolution of 60,000 in the mass range of 350–16,000 *m*/*z* with a maximum ion accumulation time set to 50 ms. The 20 most intense ions with a threshold of more than 1 × 10^6^ that had multiple charges were further fragmented by using higher energy collision dissociation at 15,000 resolution. The dynamic exclusion for higher energy collision dissociation fragmentation was 30 s. The maximum time for fragmented ion accumulation was 30 ms, with a target value of 2.50 × 10^3^, the normalized collision energy at 28%, and an isolation width of 1.6. During the acquisition, the ion transfer tube temperature was set at 160 °C, data were acquired in data-dependent acquisition mode, and the total run time was 75 min.

### Identification, quantification and statistical analysis of LC–MS data

Raw LC–MS data files from Q-Exactive HF were converted to .mgf files using Proteo Wizard MS covertgui 64 bit and analysed using Mascot v2.4. The annotated protein sequence for *P. falciparum* was downloaded from https://plasmodb.org (Release 51). Trypsin was set as the enzyme of choice with maximum missed cleavage 1, fixed modification carbamidomethyl (K), variable modification, deamidation and oxidation of N, Q and M, respectively, with the peptide and fragment mass tolerance at 0.6 Da.

### ChIP–qPCR

After de-cross-linking, ChIP DNA was purified using the Zymo ChIP DNA kit and quantified by Qubit HS DNA assay. The purified ChIP DNA was first diluted 20-fold in elution buffer and then analysed by qPCR using the CFX-96 Bio-Rad system. All ChIP primers used (Supplementary Data 8) were first checked using genomic DNA to determine specificity (based on a single peak in the melting curve) and efficiency. ChIP–qPCR data were analysed using the ΔΔCt method. *pfap2-p* ChIP–qPCR results are expressed as a percentage of input. Three biological replicates of both samples and negative control (mock IP using IgG) were used for ChIP–qPCR experiments.

### Flow cytometry using pooled human serum

iRBCs with trophozoite stage parasites from cycle 1, treated with DMSO or rapamycin in cycle 0 to disrupt the first peak of *pfap2-p* expression, were washed thrice with PBS supplemented with 0.1% BSA. iRBCs were either untreated or treated with pooled human immune serum^21^. When untreated, the same volume of 0.1% BSA in PBS was added and incubated for 30 min at room temperature. Cells were washed thrice with 0.1% BSA in PBS, and all the samples were treated with SYBR Green (1×) and mouse anti-human IgG conjugated with Alexa Fluor 647 (1:100 dilution, from BioLegend) for 30 min at room temperature. After incubation, samples were washed thrice again with 0.1% BSA in PBS and analysed on an BD LSR Fortessa flow cytometer (BD Biosciences). Data were analysed using FlowJo v9 software. Microsoft Excel (2016) was used to determine exact *P* values for different comparisons.

### In situ Hi-C

Parasites were cross-linked using 1.25% formaldehyde in warm 1× PBS for 25 min at 37 °C with rotation. Glycine was then added to a final concentration of 150 mM to quench the formaldehyde and incubated for 15 min at 37 °C and 15 min at 4 °C, both with rotation. Following centrifugation at 660*g* for 15 min at 4 °C, the pellet was resuspended in five volumes of ice-cold 1× PBS and incubated for 10 min at 4 °C with rotation. After another centrifugation at 660*g* for 15 min at 4 °C the pellet was resuspended in 20 ml ice-cold 1× PBS. Several more washes in cold 1× PBS were used to clear cellular debris before resuspending in 1 ml 1× PBS and separated into multiple 1.5 ml tubes at a concentration of 1 × 10^8^ parasites per tube. The tubes were flash frozen in liquid nitrogen and stored at 80 °C before continuing with the rest of the in situ Hi-C protocol^66^ using MboI restriction enzyme, with modifications to the standard protocol^67^. The final Hi-C libraries were sequenced using the Illumina NovaSeq 6000 using the S4 300 cycle flow cell for paired-end read libraries.

### Hi-C data processing and analysis

Paired-end Hi-C library reads were processed using HiC-Pro^68^ with default parameters and mapping at 10 kb resolution to the *P. falciparum* genome (release-50, https://plasmodb.org). The ICED-normalized interaction matrices output by HiC-Pro v 3.1.0 were interaction counts per million normalized before generating interaction heatmaps. All intra-bin contacts and contacts within a two-bin distance were set to 0 to enhance visualization and the colour map per chromosome was scaled on the basis of the minimum number of interactions in the highest 10% of interacting bins to aid in comparison between samples. Interaction matrices for the replicates were merged and differential interactions were identified by calculating the log_2_ fold change between the wild type and AP2-KO at each timepoint. Coordinate matrices generated by PASTIS v0.4.0 (ref. ^69^) were visualized as three-dimensional chromatin models using ChimeraX v1.2.5 (ref. ^70^).

### Reporting summary

Further information on research design is available in the Nature Portfolio Reporting Summary linked to this article.

### Supplementary information

Supplementary Information Supplementary discussion, references and legends for Supplementary Data 1–8.

Reporting Summary

Supplementary Data 1 List of differentially expressed genes at 16 h.p.i. and 40 h.p.i. after disruption of first and second peak of *pfap2-p* expression, respectively.

Supplementary Data 2 GO enrichment analysis of differentially expressed genes at 16 h.p.i. and 40 h.p.i. in *Δpfap2-p* parasites.

Supplementary Data 3 ChIP–seq peaks identified at 16 h.p.i. and 40 h.p.i.

Supplementary Data 4 GO enrichment analysis of genes whose promoter regions or gene body were bound by PfAP2-P at 16 h.p.i. and 40 h.p.i.

Supplementary Data 5 Comparison of PfAP2-P ChIP–seq peaks detected at 16 h.p.i. and 40 h.p.i. in this study with peaks detected in ring, trophozoite and schizont stages reported by Shang et al.^26^.

Supplementary Data 6 PfAP2-P associated proteins identified at 16 h.p.i. and 40 h.p.i. using immunoprecipitation and mass spectrometry.

Supplementary Data 7 Status of histone marks H3K4me3, H3K9ac and H3K9me3 in RAPA and mock-treated control parasites at 16 h.p.i. and 40 h.p.i.

Supplementary Data 8 Oligonucleotides used in this study.

### Source data

Source Data Fig. 1 Unprocessed western blots.

Source Data Fig. 2 Unprocessed western blots.

## Data Availability

The datasets generated in this study are available in the following databases. RNA-seq data: NCBI BioProject accession no. GSE190342; scRNA-seq: NCBI BioProject accession no. GSE191025; AP2-P ChIP–seq data: NCBI BioProject accession no. GSE190497; histone marks ChIP–seq data: NCBI BioProject accession no. GSE230206; proteomics data: Pride accession number no. PXD030308; Hi-C data: ENA BioProject accession no. PRJNA847684. The bulk RNA-seq, scRNA-seq and ChIP–seq datasets have been added under the super series GSE190519. Source data are provided with this paper.

## References

[1] Toenhake CG (2018). Chromatin accessibility-based characterization of the gene regulatory network underlying plasmodium falciparum blood-stage development. Cell Host Microbe.

[2] Cortes A, Deitsch KW (2017). Malaria epigenetics. Cold Spring Harb. Perspect. Med..

[3] Iwanaga S, Kaneko I, Kato T, Yuda M (2012). Identification of an AP2-family protein that is critical for malaria liver stage development. PLoS ONE.

[4] Kafsack BF (2014). A transcriptional switch underlies commitment to sexual development in malaria parasites. Nature.

[5] Painter HJ, Campbell TL, Llinas M (2011). The Apicomplexan AP2 family: integral factors regulating *Plasmodium* development. Mol. Biochem. Parasitol..

[6] Tinto-Font E (2021). A heat-shock response regulated by the PfAP2-HS transcription factor protects human malaria parasites from febrile temperatures. Nat. Microbiol..

[7] Yuda M, Iwanaga S, Shigenobu S, Kato T, Kaneko I (2010). Transcription factor AP2-Sp and its target genes in malarial sporozoites. Mol. Microbiol..

[8] Yuda M (2009). Identification of a transcription factor in the mosquito-invasive stage of malaria parasites. Mol. Microbiol..

[9] Collins CR (2013). Malaria parasite cGMP-dependent protein kinase regulates blood stage merozoite secretory organelle discharge and egress. PLoS Pathog..

[10] Knuepfer E, Napiorkowska M, van Ooij C, Holder AA (2017). Generating conditional gene knockouts in *Plasmodium*—a toolkit to produce stable DiCre recombinase-expressing parasite lines using CRISPR/Cas9. Sci. Rep..

[11] Subudhi AK (2020). Malaria parasites regulate intra-erythrocytic development duration via serpentine receptor 10 to coordinate with host rhythms. Nat. Commun..

[12] Gomes AR (2015). A genome-scale vector resource enables high-throughput reverse genetic screening in a malaria parasite. Cell Host Microbe.

[13] Zhang M (2018). Uncovering the essential genes of the human malaria parasite *Plasmodium falciparum* by saturation mutagenesis. Science.

[14] Thomas JA (2018). A protease cascade regulates release of the human malaria parasite *Plasmodium falciparum* from host red blood cells. Nat. Microbiol..

[15] Scherf A, Lopez-Rubio JJ, Riviere L (2008). Antigenic variation in *Plasmodium falciparum*. Annu. Rev. Microbiol..

[16] Baker DA (2017). Cyclic nucleotide signalling in malaria parasites. Open Biol..

[17] Singh S, Chitnis CE (2017). Molecular signaling involved in entry and exit of malaria parasites from host erythrocytes. Cold Spring Harb. Perspect. Med..

[18] Sargeant TJ (2006). Lineage-specific expansion of proteins exported to erythrocytes in malaria parasites. Genome Biol..

[19] Siddiqui G, Proellochs NI, Cooke BM (2020). Identification of essential exported *Plasmodium falciparum* protein kinases in malaria-infected red blood cells. Br. J. Haematol..

[20] Howick VM (2019). The Malaria Cell Atlas: single parasite transcriptomes across the complete *Plasmodium* life cycle. Science.

[21] Taylor TE (1992). Intravenous immunoglobulin in the treatment of paediatric cerebral malaria. Clin. Exp. Immunol..

[22] Chan JA, Fowkes FJ, Beeson JG (2014). Surface antigens of *Plasmodium falciparum*-infected erythrocytes as immune targets and malaria vaccine candidates. Cell. Mol. Life Sci..

[23] Gulati S (2015). Profiling the essential nature of lipid metabolism in asexual blood and gametocyte stages of *Plasmodium falciparum*. Cell Host Microbe.

[24] Josling GA, Llinas M (2015). Sexual development in *Plasmodium* parasites: knowing when it’s time to commit. Nat. Rev. Microbiol..

[25] Poran A (2017). Single-cell RNA sequencing reveals a signature of sexual commitment in malaria parasites. Nature.

[26] Shang X (2022). Genome-wide landscape of ApiAP2 transcription factors reveals a heterochromatin-associated regulatory network during *Plasmodium falciparum* blood-stage development. Nucleic Acids Res..

[27] Santos JM (2017). Red blood cell invasion by the malaria parasite is coordinated by the PfAP2-I transcription factor. Cell Host Microbe.

[28] Josling GA (2020). Dissecting the role of PfAP2-G in malaria gametocytogenesis. Nat. Commun..

[29] Lopez-Rubio JJ (2007). 5' flanking region of var genes nucleate histone modification patterns linked to phenotypic inheritance of virulence traits in malaria parasites. Mol. Microbiol..

[30] Flueck C (2009). *Plasmodium falciparum* heterochromatin protein 1 marks genomic loci linked to phenotypic variation of exported virulence factors. PLoS Pathog..

[31] Perez-Toledo K (2009). *Plasmodium falciparum* heterochromatin protein 1 binds to tri-methylated histone 3 lysine 9 and is linked to mutually exclusive expression of *var* genes. Nucleic Acids Res..

[32] Fraschka SA (2018). Comparative heterochromatin profiling reveals conserved and unique epigenome signatures linked to adaptation and development of malaria parasites. Cell Host Microbe.

[33] Zanghi G (2018). A specific PfEMP1 is expressed in *P. falciparum* sporozoites and plays a role in hepatocyte infection. Cell Rep..

[34] Meerstein-Kessel L (2021). Novel insights from the *Plasmodium falciparum* sporozoite-specific proteome by probabilistic integration of 26 studies. PLoS Comput. Biol..

[35] Moon RW (2013). Adaptation of the genetically tractable malaria pathogen *Plasmodium knowlesi* to continuous culture in human erythrocytes. Proc. Natl Acad. Sci. USA.

[36] Jones ML (2016). A versatile strategy for rapid conditional genome engineering using loxP sites in a small synthetic intron in *Plasmodium falciparum*. Sci. Rep..

[37] FastQC (2018). *Babraham Bioinformatics* http://www.bioinformatics.babraham.ac.uk/projects/fastqc

[38] Bolger AM, Lohse M, Usadel B (2014). Trimmomatic: a flexible trimmer for Illumina sequence data. Bioinformatics.

[39] Kim D, Langmead B, Salzberg SL (2015). HISAT: a fast spliced aligner with low memory requirements. Nat. Methods.

[40] Liao Y, Smyth GK, Shi W (2014). featureCounts: an efficient general purpose program for assigning sequence reads to genomic features. Bioinformatics.

[41] McCarthy DJ, Chen Y, Smyth GK (2012). Differential expression analysis of multifactor RNA-Seq experiments with respect to biological variation. Nucleic Acids Res..

[42] Ritchie ME (2015). limma powers differential expression analyses for RNA-sequencing and microarray studies. Nucleic Acids Res..

[43] Love MI, Huber W, Anders S (2014). Moderated estimation of fold change and dispersion for RNA-seq data with DESeq2. Genome Biol..

[44] Pfaffl MW (2001). A new mathematical model for relative quantification in real-time RT–PCR. Nucleic Acids Res..

[45] Jiang L (2013). PfSETvs methylation of histone H3K36 represses virulence genes in *Plasmodium falciparum*. Nature.

[46] Zeeshan M (2020). Real-time dynamics of *Plasmodium* NDC80 reveals unusual modes of chromosome segregation during parasite proliferation. J. Cell Sci..

[47] Li H (2009). The Sequence Alignment/Map format and SAMtools. Bioinformatics.

[48] Ramirez F, Dundar F, Diehl S, Gruning BA, Manke T (2014). deepTools: a flexible platform for exploring deep-sequencing data. Nucleic Acids Res..

[49] Thorvaldsdottir H, Robinson JT, Mesirov JP (2013). Integrative Genomics Viewer (IGV): high-performance genomics data visualization and exploration. Brief. Bioinform..

[50] Zhang Y (2008). Model-based analysis of ChIP-Seq (MACS). Genome Biol..

[51] Quinlan AR, Hall IM (2010). BEDTools: a flexible suite of utilities for comparing genomic features. Bioinformatics.

[52] Lun ATL (2019). EmptyDrops: distinguishing cells from empty droplets in droplet-based single-cell RNA sequencing data. Genome Biol..

[53] McCarthy DJ, Campbell KR, Lun AT, Wills QF (2017). Scater: pre-processing, quality control, normalization and visualization of single-cell RNA-seq data in R. Bioinformatics.

[54] Lun AT, McCarthy DJ, Marioni JC (2016). A step-by-step workflow for low-level analysis of single-cell RNA-seq data with Bioconductor. F1000Res.

[55] Germain PL, Lun A, Garcia Meixide C, Macnair W, Robinson MD (2021). Doublet identification in single-cell sequencing data using scDblFinder. F1000Res.

[56] scDblFinder (2021). *GitHub* https://github.com/plger/scDblFinder

[57] Hao Y (2021). Integrated analysis of multimodal single-cell data. Cell.

[58] Real E (2021). A single-cell atlas of *Plasmodium falciparum* transmission through the mosquito. Nat. Commun..

[59] Malaria Cell Atlas (2019). *GitHub* https://github.com/vhowick/MalariaCellAtlas/blob/master/Expression_Matrices/10X/pf10xIDC/pf10xIDC_pheno.csv

[60] Aran D (2019). Reference-based analysis of lung single-cell sequencing reveals a transitional profibrotic macrophage. Nat. Immunol..

[61] de Kanter JK, Lijnzaad P, Candelli T, Margaritis T, Holstege FCP (2019). CHETAH: a selective, hierarchical cell type identification method for single-cell RNA sequencing. Nucleic Acids Res..

[62] Introduction to scRNA-seq integration. *Satija Lab* https://satijalab.org/seurat/articles/integration_introduction.html#integration-goals-1 (2023).

[63] Bailey TL (2011). DREME: motif discovery in transcription factor ChIP–seq data. Bioinformatics.

[64] Gupta S, Stamatoyannopoulos JA, Bailey TL, Noble WS (2007). Quantifying similarity between motifs. Genome Biol..

[65] Campbell TL, De Silva EK, Olszewski KL, Elemento O, Llinas M (2010). Identification and genome-wide prediction of DNA binding specificities for the ApiAP2 family of regulators from the malaria parasite. PLoS Pathog..

[66] Rao SS (2014). A 3D map of the human genome at kilobase resolution reveals principles of chromatin looping. Cell.

[67] Gupta MK, Lenz T, Le Roch KG (2021). Chromosomes conformation capture coupled with next-generation sequencing (Hi-C) in *Plasmodium falciparum*. Methods Mol. Biol..

[68] Servant N (2015). HiC-Pro: an optimized and flexible pipeline for Hi-C data processing. Genome Biol..

[69] Varoquaux N, Ay F, Noble WS, Vert JP (2014). A statistical approach for inferring the 3D structure of the genome. Bioinformatics.

[70] Goddard TD (2018). UCSF ChimeraX: meeting modern challenges in visualization and analysis. Protein Sci..

