# Supplementary material for: DNA-binding protein PfAP2-P regulates parasite pathogenesis during malaria parasite blood stages
Source: Nat Microbiol. 2023 Oct 26;8(11):2154–69. doi: 10.1038/s41564-023-01497-6 (PMC10627835; doi:10.1038/s41564-023-01497-6)
Supplement: Supplementary file 1 — Supplementary discussion, references and legends for Supplementary Data 1–8. [file 41564_2023_1497_MOESM1_ESM.pdf]

nature microbiology

Article

https://doi.org/10.1038/s41564-023-01497-6

DNA-binding protein PfAP2-P regulates parasite pathogenesis during malaria parasite blood stages

In the format provided by the authors and unedited

This file contains supplementary discussion, references and legends for supplementary data 1-8.

Supplementary Discussion

Previous functional studies that have tried to decipher *var* gene regulation have implicated many chromatin-associated proteins[1]. One such protein is heterochromatin protein 1 (HP1) that facilitates silencing of all but one *var* gene by binding to trimethylated H3K9 (H3K9me3 mark)[2-5]. Other chromatin-associated proteins that also have been implicated in *var* gene regulation include silent information regulator 2a and 2b-class III-type histone deacetylases (sir2a and 2b)[6], histone deacetylase 2-a class II-type histone deacetylase (HDAC2)[7] and histone methyltransferases SET2 and SET10[8,9]. The promoters of active *var* genes were shown to be enriched with histone modifications like H3K9ac and H3K4me3 and histone variants such as H2A.Z and H2B.Z[10,11]. While these chromatin-associated proteins have been shown to participate in *var* gene regulation, in this study a few novel chromatin remodelers and histone modifiers were identified in complex with PfAP2-P (**Fig. 4h,i and Supplementary Data 6**), supporting the idea that PfAP2-P recruits these epigenetic regulators to regulate *var* and other virulence gene expression through chromatin organization. These proteins include PfMORC, EELM2 domain-containing proteins, imitation switch (ISW1), HDAC1-a class I-type histone deacetylase and chromodomain-helicase-DNA binding domain-containing proteins. Identification of PfEELM2 and PfMORC bound to PfAP2-P is consistent with results from a *P. berghei* study in which PBANKA_0939100, the ortholog of PfAP2-P, was shown to interact with PbEELM2 (PBANKA_1234600) and PbMORC (PBANKA_1331400), in schizonts[12]. EELM2 and MORC proteins are often associated with histone deacetylase in a complex with chromatin remodelling activities associated with gene suppression[13]. MORC proteins have the capability to topologically constrain DNA to facilitate gene silencing via chromatin compaction[14,15]. Recently *Toxoplasma gondii* MORC was shown to be a transcriptional repressor of sexual commitment when interacting with histone deacetylase (HDAC3) and AP2 DNA-binding proteins[16]. In this study, we observed no association of PfHDAC3 with PfAP2-P. However, we observed association of PfHDAC1 with PfAP2-P at both 16 and 40 h.p.i. (**Fig. 4h,i and Supplementary Data 6**). In previous work[17], we showed that PfMORC and PfHDAC1 have two expression peaks in the IDC, coincident with those of PfAP2-P (Pearson correlation > 0.7).

It is therefore possible that PfAP2-P interacts with PfMORC and PfHDAC1 to repress *var* gene expression.

We have observed weak or no association of HP1 with PfAP2-P at 16 h.p.i and 40 h.p.i. respectively and no association of PfSIR2A or 2B with PfAP2-P at both 16 and 40 h.p.i (**Supplementary Data 6**). We also observed no depletion of the H3K9me3 mark or enrichment of H3K9ac and H3K4me3 marks in *Δpfap2-p* parasites, which are linked to HP1, PfSIR2A and 2B involvement (**Extended Data Fig. 9**). Therefore, we suggest that PfAP2-P together with other chromatin regulators and histone modifiers act independent of HP1 and the reported histone modifications (i.e. H3K9me3, H3K9ac and H3K4me3) to either suppress and/or activate *var*, other antigenic variant protein genes and sub-telomeric heterochromatin-associated genes. In future, characterization of each PfAP2-P associated protein will help reveal the mechanistic basis of complex gene-regulatory processes behind *var* gene regulation. As we identified known transcriptional activators such as PfSET10, PfSET6, and PfISWI interacting with PfAP2-P, we propose that PfAP2-P recruits histone modifiers and chromatin remodelers to alter cis-chromatin structure, leading to either gene activation or repression. This idea is consistent with PfAP2-P acting as both activator (of some genes associated with antigenic variation, host cell modification, egress and invasion) and repressor (of *var* and gametocytogenesis-associated genes).

In a previous study, it was demonstrated that PfAP2-I and PfAP2-G bind to the same region of many invasion-associated gene promoters[18]. Our ChIP-seq data and motif enrichment analysis indicate that PfAP2-P also binds to the same genomic region of many of these genes (**Extended Data Fig. 7d,e**). This raises the possibility of a complex system of a combinatorial gene regulation, important for both asexual growth and sexual commitment. For example, a combinatorial binding of transcription factors to the same promoter has been proposed as a mechanism to increase specificity and facilitate fine-tuning[19], and the binding of PfAP2-P and other PfAP2 transcription factors to the same sites is consistent with such a mechanism. A recent study[20], suggested that ApiAP2 binding is regulated by multiple other ApiAP2s. Two examples are *pfap2-hc* and PF3D7_0613800 (an uncharacterized ApiAP2), which are regulated by at least 8 ApiAP2s

including themselves, consistent with a role for Api-AP2-mediated combinatorial gene regulation in malaria parasites.

Disruption of PfAP2-P function at 40 h.p.i. was sufficient to down-regulate genes associated with merozoite development, egress and invasion processes (**Fig. 2e, f and Supplementary Data 1**). Using immunoprecipitation and mass spectrometry, we and other have demonstrated that PfAP2-P forms complexes with other Api-AP2s and chromatin-associated proteins, indicating that PfAP2-P-regulated genes may be under the combinatorial control of multiple regulatory factors. We also showed that PfAP2-P and PfAP2-I[21] bind to the same promoter regions of many invasion and egress-associated genes. Our results from RNA-seq, ChIP-seq and IP-mass spectrometry suggest that there is a positive feedback loop-based transcriptional regulatory network between these two transcription factors (**Extended Data Fig. 7a-c**). We observed over-expression of many early gametocyte marker genes in *Δpfap2-p* parasites at both 16 and 40 h.p.i (**Extended Data Fig. 5b**). This up-regulation of sexual genes may be explained by the decrease in heterochromatin clustering as well as a direct interaction with critical AP2 transcription factors. Overall, we suggest that as an essential positive regulator of asexual growth, PfAP2-P has evolved to diminish sexual commitment by repressing early gametocyte marker genes necessary for sexual conversion.

In addition to enrichment of egress and invasion-associated genes, we identified many genes encoding hypothetical proteins of unknown function in the list of most down-regulated genes at 40 h.p.i. (n=50). Based on the concept of 'guilt-by-association'[22], we suggest that these hypothetical proteins are previously unidentified components of the egress and invasion pathways. In fact, four of these genes (Pf3D7_1014100, Pf3D7_0210600, PF3D7_0308300, PF3D7_0507400) encode proteins recently shown to be important in invasion and egress[23-26]. Further studies will be required to identify downstream regulator(s) of these invasion and egress-associated genes. The functional characterization of PfAP2-P-associated proteins will elucidate their specific roles in chromatin remodeling, histone modification, and transcription.

IFA analysis of PfAP2-P protein at different time points suggested that it is present throughout asexual development despite two waves of transcription (**Extended Data Fig. 2d**). Perhaps

PfAP2-P activity is also regulated by post translational modifications (PTMs), which may confer stability or modify protein and DNA binding and cellular location[27].

Signal transduction pathways mediated by protein kinases and phosphatases have crucial roles in the parasite life cycle[28,29] including during merozoite egress and erythrocyte invasion stages, where there are substantial differences in the phosphoproteomes of intracellular schizonts and extracellular merozoites[30]. Many of the proteins phosphorylated in merozoites may participate in egress, movement, and invasion, and are phosphorylated by kinases such as PKG, PKA, CDPK1, and CDPK5. Genes encoding these kinases were down-regulated in *Δpfap2-p* parasites at 40 h.p.i., suggesting a significant role for PfAP2-P in establishing the phosphorylation mechanisms regulating these pathogenic processes (**Extended Data Fig. 4c**).

FIKK kinases are orphan kinases restricted to apicomplexan parasites, with at least one single FIKK gene present in all *Plasmodium* genomes. However, in species of the Laverania clade, the family has been expanded to between 18 and 26 distinct members, with 21 genes in the *fikk* family of *P. falciparum* [31,32]. Eighteen of these genes code for proteins with a putative signal sequence and suggested to be exported into the iRBC to mediate parasite-induced modification central to pathogenesis[33]. Other roles are also possible, for example it was shown that FIKK3 co-localizes with PfRAMA in the rhoptry bulb[34]. A total of 12 *fikk* genes, including *fikk3,* were identified as down-regulated in *Δpfap2-p* parasites at 16 and 40 h.p.i., consistent with a role in host cell remodeling and invasion (**Extended Data Fig. 4c**).

Increased expression of many known and putative gametocyte-marker genes was observed in *Δpfap2-p* parasites at both 16 and 40 h.p.i. (**Extended Data Fig. 5b**), including genes encoding recently identified putative transcriptional regulators of gametocytogenesis such as lysine-specific demethylase (LSD2, a putative histone demethylase), AP2-O4, AP2-G3, and AP2 (PF3D7_1139300)[35,36]. LSD2 and AP2 were identified as potential regulators driving the expression of genes for gametocyte development in committed schizonts[37]. Other up-regulated genes include PfMDV-1 and an mRNA binding protein (PfPuf2) important in male and female gametocyte development, respectively[38,39]. The data suggest that PfAP2-P inhibits commitment to sexual stage development by acting through an indirect regulator as we observed no binding of

PfAP2-P to the promoters of these genes. In parasites with *ap2-p* truncated at 40 h.p.i., we identified *ap2-g* as down-regulated by ~ 2-fold compared to mock-treated control parasites. While AP2-G was identified to be down-regulated, AP2-G3 which is thought to act upstream of AP2-G[36] was found to be strongly up-regulated in AP2-P truncated parasites at 40 h.p.i. AP2-G3 localizes to nucleus as well as cytoplasm and its knockout leads to loss of gametocyte formation. Based on these observations, it was hypothesized that AP2-G3 possibly senses the environmental signal, and then activates the downstream AP2-G to initiate the gametocyte differentiation process[40]. As mentioned above, we have observed strong up-regulation of AP2-G3 and down-regulation of AP2-G. We have not observed differential expression of gametocyte development protein 1(GDV1) in *ap2-p* truncated parasites at 40 h.p.i which is an upstream activator of sexual commitment[41]. Therefore, we speculate that the up-regulation of early gametocyte marker genes observed in this study might be regulated by AP2-G3 upon decrease in heterochromatin clustering in *Δpfap2-p* parasites through an alternate mode of regulation not involving AP2-G and GDV1.

Although conditional deletion of exon 2 that encodes the AP2 domain and NLS resulted in the phenotypes we described, any residual function of the truncated protein encoded by exon 1 has not been explored. Such function would likely be mediated in the cytoplasm in the absence of translocation to the nucleus.

Legends

Supplementary Data 1 | List of differentially expressed genes at 16 h.p.i. and 40 h.p.i. after disruption of first and second peak of *pfap2-p* expression, respectively.
Supplementary Data 2 | Gene ontology enrichment analysis of differentially expressed genes at 16 h.p.i. and 40 h.p.i. in *Δpfap2-p* parasites.
Supplementary Data 3 | ChIP-seq peaks identified at 16 h.p.i. and 40 h.p.i.
Supplementary Data 4 | Gene ontology enrichment analysis of genes whose promoter regions or gene body were bound by PfAP2-P at 16 h.p.i. and 40 h.p.i.
Supplementary Data 5 | Comparison of PfAP2-P ChIP-seq peaks detected at 16 h.p.i. and 40 h.p.i. in this study with peaks detected in ring, trophozoite and schizont stages reported by Shang et al[3].
Supplementary Data 6 | PfAP2-P associated proteins identified at 16 and 40 h.p.i. using immunoprecipitation and mass spectrometry.
Supplementary Data 7 | Status of histone marks H3K4me3, H3K9ac and H3K9me3 in RAPA and mock-treated control parasites at 16 and 40 h.p.i.

Supplementary Data 8 | Oligonucleotides used in this study

References

1 Bryant, J. M. *et al.* Exploring the virulence gene interactome with CRISPR/dCas9 in the human malaria parasite. *Mol Syst Biol* **16**, e9569, doi:10.15252/msb.20209569 (2020).

2 Flueck, C. *et al.* Plasmodium falciparum heterochromatin protein 1 marks genomic loci linked to phenotypic variation of exported virulence factors. *PLoS Pathog* **5**, e1000569, doi:10.1371/journal.ppat.1000569 (2009).

3 Perez-Toledo, K. *et al.* Plasmodium falciparum heterochromatin protein 1 binds to tri-methylated histone 3 lysine 9 and is linked to mutually exclusive expression of var genes. *Nucleic Acids Res* **37**, 2596-2606, doi:10.1093/nar/gkp115 (2009).

4 Fraschka, S. A. *et al.* Comparative Heterochromatin Profiling Reveals Conserved and Unique Epigenome Signatures Linked to Adaptation and Development of Malaria Parasites. *Cell Host Microbe* **23**, 407-420 e408, doi:10.1016/j.chom.2018.01.008 (2018).

5 Zanghi, G. *et al.* A Specific PfEMP1 Is Expressed in P. falciparum Sporozoites and Plays a Role in Hepatocyte Infection. *Cell Rep* **22**, 2951-2963, doi:10.1016/j.celrep.2018.02.075 (2018).

6 Tonkin, C. J. *et al.* Sir2 paralogues cooperate to regulate virulence genes and antigenic variation in Plasmodium falciparum. *PLoS Biol* **7**, e84, doi:10.1371/journal.pbio.1000084 (2009).

7 Coleman, B. I. *et al.* A Plasmodium falciparum histone deacetylase regulates antigenic variation and gametocyte conversion. *Cell Host Microbe* **16**, 177-186, doi:10.1016/j.chom.2014.06.014 (2014).

8 Volz, J. C. *et al.* PfSET10, a Plasmodium falciparum methyltransferase, maintains the active var gene in a poised state during parasite division. *Cell Host Microbe* **11**, 7-18, doi:10.1016/j.chom.2011.11.011 (2012).

9 Ukaegbu, U. E. *et al.* Recruitment of PfSET2 by RNA polymerase II to variant antigen encoding loci contributes to antigenic variation in P. falciparum. *PLoS Pathog* **10**, e1003854, doi:10.1371/journal.ppat.1003854 (2014).

10 Lopez-Rubio, J. J. *et al.* 5' flanking region of var genes nucleate histone modification patterns linked to phenotypic inheritance of virulence traits in malaria parasites. *Mol Microbiol* **66**, 1296-1305, doi:10.1111/j.1365-2958.2007.06009.x (2007).

11 Petter, M. *et al.* H2A.Z and H2B.Z double-variant nucleosomes define intergenic regions and dynamically occupy var gene promoters in the malaria parasite Plasmodium falciparum. *Mol Microbiol* **87**, 1167-1182, doi:10.1111/mmi.12154 (2013).

12 Hillier, C. *et al.* Landscape of the Plasmodium Interactome Reveals Both Conserved and Species-Specific Functionality. *Cell Rep* **28**, 1635-1647 e1635, doi:10.1016/j.celrep.2019.07.019 (2019).

13 Solari, F., Bateman, A. & Ahringer, J. The Caenorhabditis elegans genes egl-27 and egr-1 are similar to MTA1, a member of a chromatin regulatory complex, and are redundantly required for embryonic patterning. *Development* **126**, 2483-2494 (1999).

14 Kim, H. *et al.* The Gene-Silencing Protein MORC-1 Topologically Entraps DNA and Forms Multimeric Assemblies to Cause DNA Compaction. *Mol Cell* **75**, 700-710 e706, doi:10.1016/j.molcel.2019.07.032 (2019).

15 Koch, A. *et al.* MORC Proteins: Novel Players in Plant and Animal Health. *Front Plant Sci* **8**, 1720, doi:10.3389/fpls.2017.01720 (2017).
16 Farhat, D. C. *et al.* A MORC-driven transcriptional switch controls Toxoplasma developmental trajectories and sexual commitment. *Nat Microbiol* **5**, 570-583, doi:10.1038/s41564-020-0674-4 (2020).
17 Subudhi, A. K. *et al.* Malaria parasites regulate intra-erythrocytic development duration via serpentine receptor 10 to coordinate with host rhythms. *Nat Commun* **11**, 2763, doi:10.1038/s41467-020-16593-y (2020).
18 Josling, G. A. *et al.* Dissecting the role of PfAP2-G in malaria gametocytogenesis. *Nat Commun* **11**, 1503, doi:10.1038/s41467-020-15026-0 (2020).
19 Reiter, F., Wienerroither, S. & Stark, A. Combinatorial function of transcription factors and cofactors. *Curr Opin Genet Dev* **43**, 73-81, doi:10.1016/j.gde.2016.12.007 (2017).
20 Shang, X. *et al.* Genome-wide landscape of ApiAP2 transcription factors reveals a heterochromatin-associated regulatory network during Plasmodium falciparum blood-stage development. *Nucleic Acids Res*, doi:10.1093/nar/gkac176 (2022).
21 Santos, J. M. *et al.* Red Blood Cell Invasion by the Malaria Parasite Is Coordinated by the PfAP2-I Transcription Factor. *Cell Host Microbe* **21**, 731-741 e710, doi:10.1016/j.chom.2017.05.006 (2017).
22 Oliver, S. Guilt-by-association goes global. *Nature* **403**, 601-603, doi:10.1038/35001165 (2000).
23 Nagaoka, H. *et al.* PfMSA180 is a novel Plasmodium falciparum vaccine antigen that interacts with human erythrocyte integrin associated protein (CD47). *Sci Rep* **9**, 5923, doi:10.1038/s41598-019-42366-9 (2019).
24 Liffner, B. *et al.* PfCERLI1 is a conserved rhoptry associated protein essential for Plasmodium falciparum merozoite invasion of erythrocytes. *Nat Commun* **11**, 1411, doi:10.1038/s41467-020-15127-w (2020).
25 Wichers, J. S. *et al.* Identification of novel inner membrane complex and apical annuli proteins of the malaria parasite Plasmodium falciparum. *Cell Microbiol* **23**, e13341, doi:10.1111/cmi.13341 (2021).
26 Tarr, S. J. *et al.* A malaria parasite subtilisin propeptide-like protein is a potent inhibitor of the egress protease SUB1. *Biochem J* **477**, 525-540, doi:10.1042/BCJ20190918 (2020).
27 Filtz, T. M., Vogel, W. K. & Leid, M. Regulation of transcription factor activity by interconnected post-translational modifications. *Trends Pharmacol Sci* **35**, 76-85, doi:10.1016/j.tips.2013.11.005 (2014).
28 Baker, D. A. *et al.* Cyclic nucleotide signalling in malaria parasites. *Open Biol* **7**, doi:10.1098/rsob.170213 (2017).
29 Singh, S. & Chitnis, C. E. Molecular Signaling Involved in Entry and Exit of Malaria Parasites from Host Erythrocytes. *Cold Spring Harb Perspect Med* **7**, doi:10.1101/cshperspect.a026815 (2017).
30 Lasonder, E., Green, J. L., Grainger, M., Langsley, G. & Holder, A. A. Extensive differential protein phosphorylation as intraerythrocytic Plasmodium falciparum schizonts develop into extracellular invasive merozoites. *Proteomics* **15**, 2716-2729, doi:10.1002/pmic.201400508 (2015).

31 Proellocks, N. I., Coppel, R. L., Mohandas, N. & Cooke, B. M. Malaria Parasite Proteins and Their Role in Alteration of the Structure and Function of Red Blood Cells. *Adv Parasitol* **91**, 1-86, doi:10.1016/bs.apar.2015.09.002 (2016).
32 Ward, P., Equinet, L., Packer, J. & Doerig, C. Protein kinases of the human malaria parasite Plasmodium falciparum: the kinome of a divergent eukaryote. *BMC Genomics* **5**, 79, doi:10.1186/1471-2164-5-79 (2004).
33 Davies, H. *et al.* An exported kinase family mediates species-specific erythrocyte remodelling and virulence in human malaria. *Nat Microbiol* **5**, 848-863, doi:10.1038/s41564-020-0702-4 (2020).
34 Siddiqui, G., Proellochs, N. I. & Cooke, B. M. Identification of essential exported Plasmodium falciparum protein kinases in malaria-infected red blood cells. *Br J Haematol* **188**, 774-783, doi:10.1111/bjh.16219 (2020).
35 Shang, X. *et al.* A cascade of transcriptional repression determines sexual commitment and development in Plasmodium falciparum. *Nucleic Acids Res* **49**, 9264-9279, doi:10.1093/nar/gkab683 (2021).
36 Zhang, C. *et al.* Systematic CRISPR-Cas9-Mediated Modifications of Plasmodium yoelii ApiAP2 Genes Reveal Functional Insights into Parasite Development. *mBio* **8**, doi:10.1128/mBio.01986-17 (2017).
37 Poran, A. *et al.* Single-cell RNA sequencing reveals a signature of sexual commitment in malaria parasites. *Nature* **551**, 95-99, doi:10.1038/nature24280 (2017).
38 Furuya, T. *et al.* Disruption of a Plasmodium falciparum gene linked to male sexual development causes early arrest in gametocytogenesis. *Proc Natl Acad Sci U S A* **102**, 16813-16818, doi:10.1073/pnas.0501858102 (2005).
39 Miao, J. *et al.* The Puf-family RNA-binding protein PfPuf2 regulates sexual development and sex differentiation in the malaria parasite Plasmodium falciparum. *J Cell Sci* **123**, 1039-1049, doi:10.1242/jcs.059824 (2010).
40 Jeninga, M. D., Quinn, J. E. & Petter, M. ApiAP2 Transcription Factors in Apicomplexan Parasites. *Pathogens* **8**, doi:10.3390/pathogens8020047 (2019).
41 Filarsky, M. *et al.* GDV1 induces sexual commitment of malaria parasites by antagonizing HP1-dependent gene silencing. *Science* **359**, 1259-1263, doi:10.1126/science.aan6042 (2018).
