# Supplementary material for: DNA-binding protein PfAP2-P regulates parasite pathogenesis during malaria parasite blood stages
Source: Nat Microbiol. 2023 Oct 26;8(11):2154–69. doi: 10.1038/s41564-023-01497-6 (PMC10627835; doi:10.1038/s41564-023-01497-6)
Supplement: Supplementary file 2 — Reporting Summary [file 41564_2023_1497_MOESM2_ESM.pdf]

nature portfolio

Corresponding author(s): Anthony A. Holder; Arnab Pain

Last updated by author(s): Aug 31, 2023

Reporting Summary

Nature Portfolio wishes to improve the reproducibility of the work that we publish. This form provides structure for consistency and transparency in reporting. For further information on Nature Portfolio policies, see our Editorial Policies and the Editorial Policy Checklist.

Statistics

For all statistical analyses, confirm that the following items are present in the figure legend, table legend, main text, or Methods section.

n/a	Confirmed	
☐	☒	The exact sample size (*n*) for each experimental group/condition, given as a discrete number and unit of measurement
☐	☒	A statement on whether measurements were taken from distinct samples or whether the same sample was measured repeatedly
☐	☒	The statistical test(s) used AND whether they are one- or two-sided<br>*Only common tests should be described solely by name; describe more complex techniques in the Methods section.*
☒	☐	A description of all covariates tested
☒	☐	A description of any assumptions or corrections, such as tests of normality and adjustment for multiple comparisons
☐	☒	A full description of the statistical parameters including central tendency (e.g. means) or other basic estimates (e.g. regression coefficient) AND variation (e.g. standard deviation) or associated estimates of uncertainty (e.g. confidence intervals)
☐	☒	For null hypothesis testing, the test statistic (e.g. *F*, *t*, *r*) with confidence intervals, effect sizes, degrees of freedom and *P* value noted<br>*Give P values as exact values whenever suitable.*
☒	☐	For Bayesian analysis, information on the choice of priors and Markov chain Monte Carlo settings
☒	☐	For hierarchical and complex designs, identification of the appropriate level for tests and full reporting of outcomes
☒	☐	Estimates of effect sizes (e.g. Cohen's *d*, Pearson's *r*), indicating how they were calculated

Our web collection on statistics for biologists contains articles on many of the points above.

Software and code

Policy information about availability of computer code

Data collection	RNA-seq, ChIP-seq, and Hi-C libraries were sequenced on an Illumina HiSeq4000 or Novaseq 6000 platform. Data were collected in FastQ format. MS/MS data were acquired using Q-Exactive HF mass spectrometer (Thermo Fisher Scientific).
Data analysis	Microsoft Excel (2016)<br>FASTQC (0.11.8)<br>trimmomatic (0.33)<br>Hisat2(2.1.0)<br>DeSeq2 (1.38.3)<br>samtools (1.8)<br>R 3.6.3<br>RStudio (1.3.1073)<br>MACS (1.4.2)<br>deeptools(3.3.1)<br>bedtools(2.29.0)<br>Homer's annotatePeaks.pl (3.2.1)<br>DREME server (https://meme-suite.org/meme/doc/dreme.html)<br>Tomtom (https://meme-suite.org/meme/tools/tomtom)<br>Intergartive Genome Browser (2.4.16)<br>cellranger(5.0.1)<br>Mascot v2.4<br>MaxQuant version 1.6.2.10.<br>Adobe Photoshop software 2021-23<br>Graphpad Prism 9<br>HiC-Pro (3.1.0)

Pastis (0.4.0)
UCSF ChimeraX (1.2.5)
SingleR package v1.6.1
dropletUtils v1.12.3
scuttle package v1.2.1
Scran R package v1.20.1
CHETAH R package v1.8.0
Seurat (4.1.1)

For manuscripts utilizing custom algorithms or software that are central to the research but not yet described in published literature, software must be made available to editors and reviewers. We strongly encourage code deposition in a community repository (e.g. GitHub). See the Nature Portfolio guidelines for submitting code & software for further information.

Data

Policy information about availability of data

All manuscripts must include a data availability statement. This statement should provide the following information, where applicable:

- Accession codes, unique identifiers, or web links for publicly available datasets
- A description of any restrictions on data availability
- For clinical datasets or third party data, please ensure that the statement adheres to our policy

PlasmoDB (Release 51; https://plasmodb.org)
The phenotypic data for MCA was obtained from https://github.com/vhowick/MalariaCellAtlas/blob/master/Expression_Matrices/10X/pf10xIDC/pf10xIDC_pheno.csv
The data sets generated in this study are available in the following databases:
• RNA-seq data: NCBI BioProject accession # GSE190342
• ScRNA-seq: NCBI BioProject accession # GSE191025
• AP2-P ChIP-seq data: NCBI BioProject accession # GSE 190497
• Histone marks ChIP-seq data: NCBI BioProject accession # GSE230206
• Proteomics data: Pride accession number # PXD030308.
• Hi-C data: ENA BioProject accession number #PRJNA847684
The bulk RNA-seq, ScRNA-seq and ChIP-Seq datasets have been added under the super series GSE190519

Field-specific reporting

Please select the one below that is the best fit for your research. If you are not sure, read the appropriate sections before making your selection.

☒ Life sciences ☐ Behavioural & social sciences ☐ Ecological, evolutionary & environmental sciences

For a reference copy of the document with all sections, see nature.com/documents/nr-reporting-summary-flat.pdf

Life sciences study design

All studies must disclose on these points even when the disclosure is negative.

Sample size	Sample sizes were not predetermined. All our data presented are from laboratory based investigation and does not include clinical data. As accepted standard, we have used a minimum of three biological replicates in each experiments and three technical experiments where necessary to derive statistics and conclusions.<br>All experiments were performed in biological replicates to allow for statistical analyses.
Data exclusions	No data were excluded from this study
Replication	Three biological replicates were used for all the in vitro experiments. All attempts of replication were successful
Randomization	No method of randomization was used and experiments were performed in biological replicates as stated for each experiment in the main manuscript. All corresponding treatment and mock samples were processed at the same time to minimize technical variation.
Blinding	N/A. This study did not involve animals or human research. All experiments were laboratory-based. Blinding was not suitable for any of the experiments

Reporting for specific materials, systems and methods

We require information from authors about some types of materials, experimental systems and methods used in many studies. Here, indicate whether each material, system or method listed is relevant to your study. If you are not sure if a list item applies to your research, read the appropriate section before selecting a response.

Materials & experimental systems

n/a	Involved in the study
☐	☒ Antibodies
☒	☐ Eukaryotic cell lines
☒	☐ Palaeontology and archaeology
☒	☐ Animals and other organisms
☒	☐ Human research participants
☒	☐ Clinical data
☒	☐ Dual use research of concern

Methods

n/a	Involved in the study
☐	☒ ChIP-seq
☐	☒ Flow cytometry
☒	☐ MRI-based neuroimaging

Antibodies

Antibodies used

For western blot and Immunofluorescence assay following antibodies were used: Rabbit anti-EBA175 (1:10,000), rat anti-MyoA (1:1,000), rat anti-BiP (1:1,000), rabbit anti-PTRAMP (1:4,000), rabbit anti-ARO (1:1,000), rabbit anti-AMA1 (1:10,000), and rabbit anti-SUB1 (1:1,000). Antibodies used in immunofluorescence were rabbit anti-GAP45 (1:1,000) and rabbit anti-MSP7 (1:1,000). Anti-EBA175 was obtained from MR4 (beiresources.org/MR4Home), anti-BiP was provided by Dr. Ellen Knuepfer, anti-SUB1 was generous gift from Prof. Mike Blackman (Francis Crick Institute) and anti-AMA1 was generous gift from Bart Faber and Clemens Kocken from the Primate Research center in Rinswijk. All other antibodies were generated in the Holder laboratory and are now held, and freely available, at NIBSC-CFAR (please contact cfar@nibsc.org with any enquiries). For ChIP-seq Rabbit anti HA antibody from Rabbit polyclonal anti-HA (Abcam no. ab9110, RRID:AB_307019), Rabbit IgG isotype control (Cat. No. 14-4301-82, Invitrogen, RRID:AB_2532981), Rabbit polyclonal anti H3K9me3 antibody (Cat no. 07-442, Millipore, RRID:AB_310620), Rabbit polyclonal anti H3K9ac antibody (Cat no. 07-352, Millipore, RRID:AB_310544), Rabbit polyclonal anti H3K4me3 antibody (Cat no. ab8580, Abcam, RRID:AB_306649) were used.

Validation

All the antibodies used in this study were validated for ChIP-seq, IFA or western by several groups according to their RRID numbers and sometimes by the company that own them.
Antibodies such as Rabbit anti-EBA175 , rat anti-MyoA , rat anti-BiP , rabbit anti-PTRAMP, rabbit anti-ARO , rabbit anti-AMA1, and rabbit anti-SUB1. Antibodies used in immunofluorescence were rabbit anti-GAP45 and rabbit anti-MSP7 were validated by the provider and published previously. (PMIDs: 29459732, 22479457 and)33287958)
The below antibodies were validated by the manufacturer and published previously
Rabbit polyclonal anti-HA (Abcam no. ab9110, RRID:AB_307019)
Rabbit IgG isotype control (Cat. No. 14-4301-82, Invitrogen, RRID:AB_2532981)
Rabbit polyclonal anti H3K9me3 antibody (Cat no. 07-442, Millipore, RRID:AB_310620)
Rabbit polyclonal anti H3K9ac antibody (Cat no. 07-352, Millipore, RRID:AB_310544)
Rabbit polyclonal anti H3K4me3 antibody (Cat no. ab8580, Abcam, RRID:AB_306649)

ChIP-seq

Data deposition

☒ Confirm that both raw and final processed data have been deposited in a public database such as GEO.

☒ Confirm that you have deposited or provided access to graph files (e.g. BED files) for the called peaks.

Data access links
May remain private before publication.

The data sets generated in this study are available in the following databases:

• AP2-P ChIP-seq data: NCBI BioProject accession # GSE 190497
• Histone marks ChIP-seq data: NCBI BioProject accession # GSE230206

Files in database submission

Chip_40hpi_AP2_HA_rep1_R1_001.fastq.gz
Chip_40hpi_AP2_HA_rep1_R2_001.fastq.gz
Chip_40hpi_AP2_HA_rep2_R1_001.fastq.gz
Chip_40hpi_AP2_HA_rep2_R2_001.fastq.gz
IgG_40hpi_AP2_HA_rep1_R1_001.fastq.gz
IgG_40hpi_AP2_HA_rep1_R2_001.fastq.gz
IgG_40hpi_AP2_HA_rep2_R1_001.fastq.gz
IgG_40hpi_AP2_HA_rep2_R2_001.fastq.gz
Input_40hpi_AP2_HA_rep1_R1_001.fastq.gz
Input_40hpi_AP2_HA_rep1_R2_001.fastq.gz
Input_40hpi_AP2_HA_rep2_R1_001.fastq.gz
Input_40hpi_AP2_HA_rep2_R2_001.fastq.gz
Chip_16hpi_AP2_HA_rep1_R1_001.fastq.gz
Chip_16hpi_AP2_HA_rep1_R2_001.fastq.g
Chip_16hpi_AP2_HA_rep2_R1_001.fastq.gz
Chip_16hpi_AP2_HA_rep2_R1_001.fastq.gz

IgG_16hpi_AP2_HA_rep1_R1_001.fastq.gz
IgG_16hpi_AP2_HA_rep1_R2_001.fastq.gz
Input_16hpi_AP2_HA_rep1_R1_001.fastq.gz
Input_16hpi_AP2_HA_rep1_R2_001.fastq.gz

Chip_40hpi_AP2_HA_rep1.bw
Chip_40hpi_AP2_HA_rep2.bw

Input_40hpi_AP2_HA_rep1.bw
Input_40hpi_AP2_HA_rep1.bw

IgG_40hpi_AP2_HA_rep1.bw
IgG_40hpi_AP2_HA_rep2.bw

Chip_40hpi_AP2_HAvsInput_rep1.bed
Chip_40hpi_AP2_HAvsInput_rep2.bed
Chip_40hpi_AP2_HAvsIgG_rep1.bed
Chip_40hpi_AP2_HAvsIgG_rep2.bed

Chip_16hpi_AP2_HA_rep1.bw
Chip_16hpi_AP2_HA_rep2.bw

IgG_16hpi_AP2_HA_rep1.bw

Input_16hpi_AP2_HA_rep1.bw
Input_16hpi_AP2_HA_rep2.bw

Chip_16hpi_AP2_HAvsIgG_rep1.bed
Chip_16hpi_AP2_HAvsInput_rep1.bed
Chip_16hpi_AP2_HAvsInput_rep2.bed

20D_H3K4ME3_rep1_R1_001.fastq.gz
20D_H3K4ME3_rep1_R2_001.fastq.gz
20D_H3K4ME3_rep2_R1_001.fastq.gz
20D_H3K4ME3_rep2_R2_001.fastq.gz
20D_H3K9ME3_rep1_R1_001.fastq.gz
20D_H3K9ME3_rep1_R2_001.fastq.gz
20D_H3K9ME3_rep2_R1_001.fastq.gz
20D_H3K9ME3_rep2_R2_001.fastq.gz
20D_H3K9ac_rep1_R1_001.fastq.gz
20D_H3K9ac_rep1_R2_001.fastq.gz
20D_H3K9ac_rep2_R1_001.fastq.gz
20D_H3K9ac_rep2_R2_001.fastq.gz
20R_H3K4ME3_rep1_R1_001.fastq.gz
20R_H3K4ME3_rep1_R2_001.fastq.gz
20R_H3K4ME3_rep2_R1_001.fastq.gz
20R_H3K4ME3_rep2_R2_001.fastq.gz
20R_H3K4ME3_rep3_R1_001.fastq.gz
20R_H3K4ME3_rep3_R2_001.fastq.gz
20R_H3K9ME3_rep1_R1_001.fastq.gz
20R_H3K9ME3_rep1_R2_001.fastq.gz
20R_H3K9ME3_rep2_R1_001.fastq.gz
20R_H3K9ME3_rep2_R2_001.fastq.gz
20R_H3K9ME3_rep3_R1_001.fastq.gz
20R_H3K9ME3_rep3_R2_001.fastq.gz
20R_H3K9ac_rep1_R1_001.fastq.gz
20R_H3K9ac_rep1_R2_001.fastq.gz
20R_H3K9ac_rep2_R1_001.fastq.gz
20R_H3K9ac_rep2_R2_001.fastq.gz
40D_H3K4ME3-rep2_R2_001.fastq.gz
40D_H3K4ME3_rep1_R1_001.fastq.gz
40D_H3K4ME3_rep1_R2_001.fastq.gz
40D_H3K4ME3_rep2_R1_001.fastq.gz
40D_H3K9ME3_rep1_R1_001.fastq.gz
40D_H3K9ME3_rep1_R2_001.fastq.gz
40D_H3K9ME3_rep2_R1_001.fastq.gz
40D_H3K9ME3_rep2_R2_001.fastq.gz
40D_H3K9ac_rep1_R1_001.fastq.gz
40D_H3K9ac_rep1_R2_001.fastq.gz
40D_H3K9ac_rep2_R1_001.fastq.gz
40D_H3K9ac_rep2_R2_001.fastq.gz
40R_H3K4ME3_rep1_R1_001.fastq.gz
40R_H3K4ME3_rep1_R2_001.fastq.gz
40R_H3K4ME3_rep2_R1_001.fastq.gz
40R_H3K4ME3_rep2_R2_001.fastq.gz
40R_H3K9ME3_rep1_R1_001.fastq.gz

40R_H3K9ME3_rep1_R2_001.fastq.gz
40R_H3K9ME3_rep2_R1_001.fastq.gz
40R_H3K9ME3_rep2_R2_001.fastq.gz
40R_H3K9ac_rep1_R1_001.fastq.gz
40R_H3K9ac_rep1_R2_001.fastq.gz
40R_H3K9ac_rep2_R1_001.fastq.gz
40R_H3K9ac_rep2_R2_001.fastq.gz
Input_20D_rep1_R1_001.fastq.gz
Input_20D_rep1_R2_001.fastq.gz
Input_20D_rep2_R1_001.fastq.gz
Input_20D_rep2_R2_001.fastq.gz
Input_20R_rep1_R1_001.fastq.gz
Input_20R_rep1_R2_001.fastq.gz
Input_20R_rep2_R1_001.fastq.gz
Input_20R_rep2_R2_001.fastq.gz
Input_40D_rep1_R1_001.fastq.gz
Input_40D_rep1_R2_001.fastq.gz
Input_40D_rep2_R1_001.fastq.gz
Input_40D_rep2_R2_001.fastq.gz

20D_H3K4ME3_rep1.bw
20D_H3K4ME3_rep2.bw
20D_H3K9ME3-rep2.bw
20D_H3K9ME3_rep1.bw
20D_H3K9ac_rep1.bw
20D_H3K9ac_rep2.bw

20R_H3K4ME3_rep1.bw
20R_H3K4ME3_rep2.bw
20R_H3K4ME3_rep3.bw
20R_H3K9ME3_rep1.bw
20R_H3K9ME3_rep2.bw
20R_H3K9ME3_rep3.bw
20R_H3K9ac_rep1.bw
20R_H3K9ac_rep2.bw

Input_20D_rep1.bw
Input_20D_rep2.bw

Input_20R_rep1.bw
Input_20R_rep2.bw

40D_H3K4ME3_rep1.bw
40D_H3K4ME3_rep2.bw
40D_H3K9ME3_rep1.bw
40D_H3K9ME3_rep2.bw
40D_H3K9ac_rep1.bw
40D_H3K9ac_rep2.bw
40R_H3K9ac_rep1.bw
40R_H3K9ac_rep2.bw

Input_40D_rep1.bw
Input_40R_rep2.bw

20D_H3K4ME3_rep1.bed
20D_H3K4ME3_rep2.bed
20D_H3K9ME3_rep1.bed
20D_H3K9ME3_rep2.bed
20D_H3K9ac_rep1.bed
20D_H3K9ac_rep2.bed
20R_H3K4ME3_rep1.bed
20R_H3K4ME3_rep2.bed
20R_H3K4ME3_rep3.bed
20R_H3K9ME3_rep1.bed
20R_H3K9ME3_rep2.bed
20R_H3K9ME3_rep3.bed
20R_H3K9ac_rep1.bed
20R_H3K9ac_rep2.bed

40D_H3K4ME3_rep1.bed
40D_H3K4ME3_rep2.bed
40D_H3K9ME3_rep1.bed
40D_H3K9ME3_rep2.bed
40D_H3K9ac_rep1.bed
40D_H3K9ac_rep2.bed
40R_H3K4ME3_rep1.bed

40R_H3K4ME3_rep2.bed
40R_H3K9ME3_rep1.bed
40R_H3K9ME3_rep2.bed
40R_H3K9ac_rep1.bed
40R_H3K9ac_rep2.bed

40R_H3K4ME3-rep2.bw
40R_H3K4ME3_rep1.bw
40R_H3K9ME3-rep1.bw
40R_H3K9ME3_rep2.bw

Genome browser session
(e.g. UCSC)

Not applicable

Methodology

Replicates

AP2-MRP 40 h.p.i. ChIP-2 replicates; 40 hpi input control -2 replicates; 40 hpi IgG control-2 replicates, AP2-MRP 16 h.p.i. ChIP-2 replicates; 16 hpi input-2 replicates; 16 hpi IgG control-1 replicates
Histone marks ChIP experment: 20D_H3K4ME3: 2 replicates, 20D_H3K9ac:2 replicates, 20D_H3K9ME3:3 replicates, 20R_H3K4ME3: 2 replicates, 20R_H3K9ac:2 replicates, 20R_H3K9ME3:3 replicates, 40D_H3K4ME3: 2 replicates, 40D_H3K9ac:2 replicates, 40D_H3K9ME3:2 replicates, 40R_H3K4ME3: 2 replicates, 40R_H3K9ac:2 replicates, 40R_H3K9ME3:3 replicates, Input_20D: 2 replicates, Input_20r: 2 replicates, Input_40D: 1 Replicate, Input_40R: 1replicate,

Sequencing depth

Sequencing layout: 2x150bp
Sequencing Depth for each sample ID
ID: total number of reads /uniquely mapped (P. falciparum genome V3)
Chip_40hpi_AP2_HA_rep1
Chip_40hpi_AP2_HA_rep2
Input_40hpi_AP2_HA_rep1
Input_40hpi_AP2_HA_rep2
IgG_40hpi_AP2_HA_rep1
IgG_40hpi_AP2_HA_rep2

Chip_16hpi_AP2_HA_rep1
Chip_16hpi_AP2_HA_rep2
Chip_16hpi_AP2_HA_rep3
IgG_16hpi_AP2_HA_rep1
Input_16hpi_AP2_HA_rep1
Input_16hpi_AP2_HA_rep2

20D_H3K4ME3_rep1
20D_H3K4ME3_rep2
20D_H3K9ME3-rep2
20D_H3K9ME3_rep1
20D_H3K9ac_rep1
20D_H3K9ac_rep2
20R_H3K4ME3_rep1
20R_H3K4ME3_rep2
20R_H3K4ME3_rep3
20R_H3K9ME3_rep1
20R_H3K9ME3_rep2
20R_H3K9ME3_rep3
20R_H3K9ac_rep1
20R_H3K9ac_rep2
Input_20D_rep1
Input_20D_rep2
Input_20R_rep1
Input_20R_rep2

40D_H3K4ME3_rep1
40D_H3K4ME3_rep2
40D_H3K9ME3_rep1
40D_H3K9ME3_rep2
40D_H3K9ac_rep1
40D_H3K9ac_rep2
40R_H3K4ME3-rep2
40R_H3K4ME3_rep1
40R_H3K9ME3-rep1
40R_H3K9ME3_rep2
40R_H3K9ac_rep1
40R_H3K9ac_rep2
Input_40D_rep1

Input_40R_rep2

Antibodies

Rabbit polyclonal anti-HA (Abcam no. ab9110, RRID:AB_307019)
Rabbit IgG isotype control (Cat. No. 14-4301-82, Invitrogen, RRID:AB_2532981)
Rabbit polyclonal anti H3K9me3 antibody (Cat no. 07-442, Millipore, RRID:AB_310620)
Rabbit polyclonal anti H3K9ac antibody (Cat no. 07-352, Millipore, RRID:AB_310544)
Rabbit polyclonal anti H3K4me3 antibody (Cat no. ab8580, Abcam, RRID:AB_306649)

Peak calling parameters

Low-quality reads and Illumina adaptor sequences from the read ends were removed using Trimmomatic (Bolger et al., 2014). Quality trimmed reads were aligned to the P. falciparum genome (plasmodb.org, v3, release v32) using HiSat2. Duplicate reads were removed using samtools (markdup) (Li et al., 2009). GC bias was corrected using deeptool's correctGCBias tool (Ramirez et al., 2014). For coverage plots of Api2EI 40 h.p.i. and 20 h.p.i. ChIP-seq experiments, deeptool's bamCompare tool was used to normalize the read coverage per base of the genome position (option '-bs 1') in the respective input and ChIP samples or IgG and ChIP samples to the total number of reads in each library (-- nomarmalizeUsing RPKM). Normalized input coverage or IgG coverage per bin was subtracted from the ChIP values (option – operation subtract). Coverage plots were visualized using IGV genome browser (Thorvaldsdottir et al., 2013). ChIP-Peaks (q-value cutoff < 0.05) were identified using macs2 (Zhang et al., 2008) by comparing the input with ChIP or IgG with ChIP with default settings but without prior peak modelling (option '-nomodel'), the fragment size set to 200 bp (option '-extsize 200') and the genome size (option '-g') set to 233332839. Robust common peaks between replicates were identified using bedtools 'intersect' (option –f 0.30 –r) (Quinlan and Hall, 2010). Peak annotation was carried out using Homer's annotatePeaks.pl that assigned each peak with the nearest downstream gene.

Data quality

Raw data that passed Trimmomatic Trimmomatic quality filter were used

Software

Trimmomatic v 0.38
BOWTIE2-2.3.5
macs2-2.1.1.20

Flow Cytometry

Plots

Confirm that:

☒ The axis labels state the marker and fluorochrome used (e.g. CD4-FITC).

☒ The axis scales are clearly visible. Include numbers along axes only for bottom left plot of group (a 'group' is an analysis of identical markers).

☒ All plots are contour plots with outliers or pseudocolor plots.

☒ A numerical value for number of cells or percentage (with statistics) is provided.

Methodology

Sample preparation

iRBCs with trophozoite stage parasites from cycle 1, treated with DMSO or rapamycin in cyle 0 to disrupt the first peak of PfAP2-MRP expression, were washed thrice with PBS supplemented with 0.1% BSA. iRBCS were either untreated or treated with Hiserum. When untreated, the same volume of 0.1% BSA in PBS was added and incubated for 30 mins at room temperature. Cells were washed thrice with 0.1% BSA in PBS, and all the samples were treated with sybr green (1x) and mouse anti-human IgG conjugated with Alexflour 647 (1:100 dilution, from BioLegend) for 30 mins at room temperature. After incubation, samples were washed thrice again with 0.1% BSA in PBS and analyzed on an LSR Fortessa (BD). Data were analyzed using FlowJo v9 software.

Instrument

LSR Fortessa (BD)

Software

Data were analyzed using FlowJo v9 software.

Cell population abundance

N/A. Sorting was not performed so not applicable here.

Gating strategy

Gating strategy has been provided as a supplementary figure

☒ Tick this box to confirm that a figure exemplifying the gating strategy is provided in the Supplementary Information.
