# Supplementary figures and images for: DNA-binding protein PfAP2-P regulates parasite pathogenesis during malaria parasite blood stages
Source: Nat Microbiol. 2023 Oct 26;8(11):2154–69. doi: 10.1038/s41564-023-01497-6 (PMC10627835; doi:10.1038/s41564-023-01497-6)

**Fig. 1d**

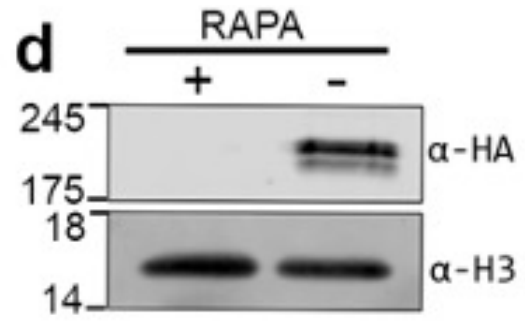

$\alpha$ -HA

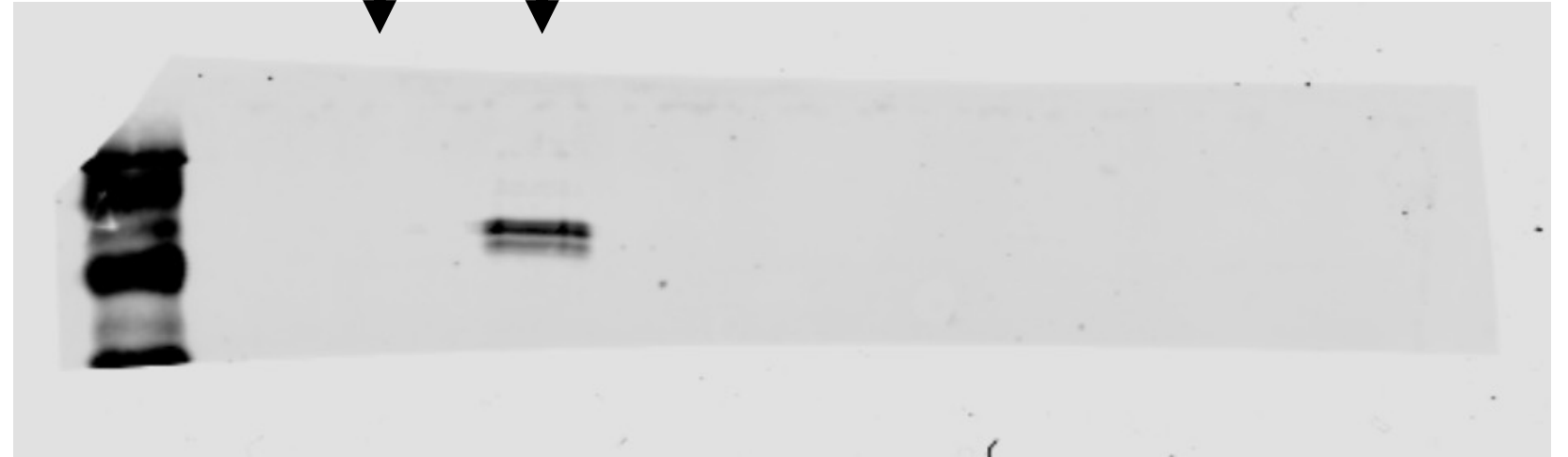

$\alpha$ -H3

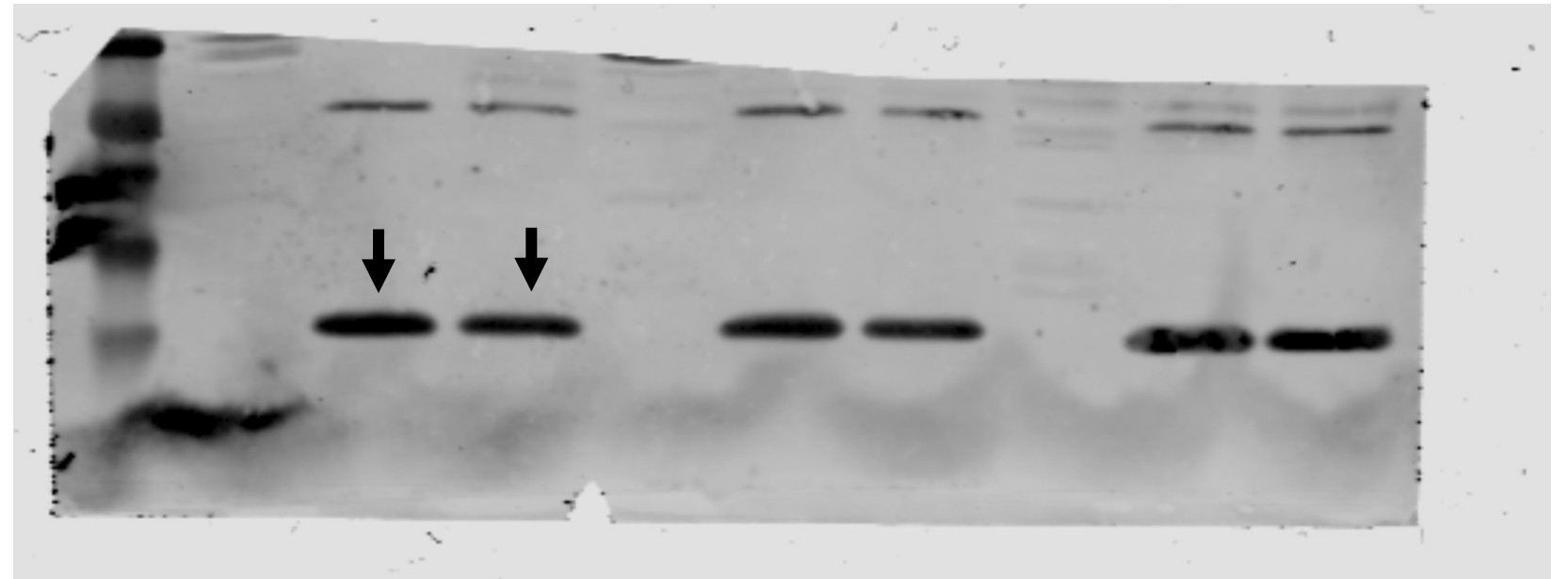

Supplement: Supplementary file 11 — Unprocessed western blots. [file 41564_2023_1497_MOESM11_ESM.pdf]
