# Supplementary material for: DNA-binding protein PfAP2-P regulates parasite pathogenesis during malaria parasite blood stages
Source: Nat Microbiol. 2023 Oct 26;8(11):2154–69. doi: 10.1038/s41564-023-01497-6 (PMC10627835; doi:10.1038/s41564-023-01497-6)

Fig. 2g

Uncropped image of EBA 175

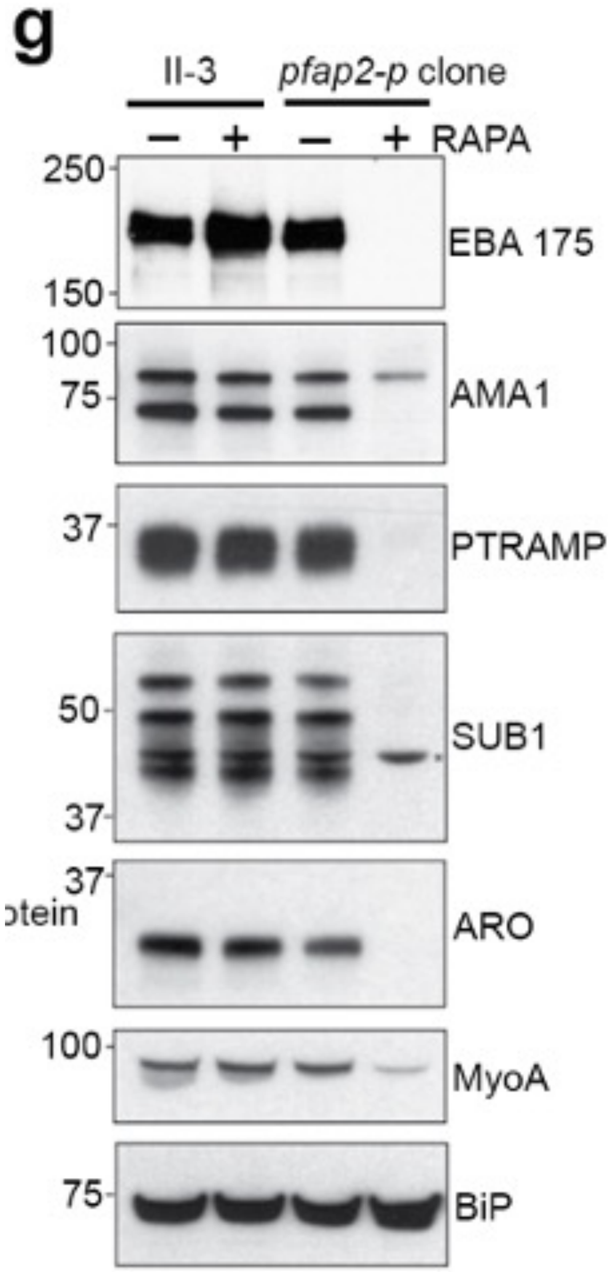

α -EBA 175

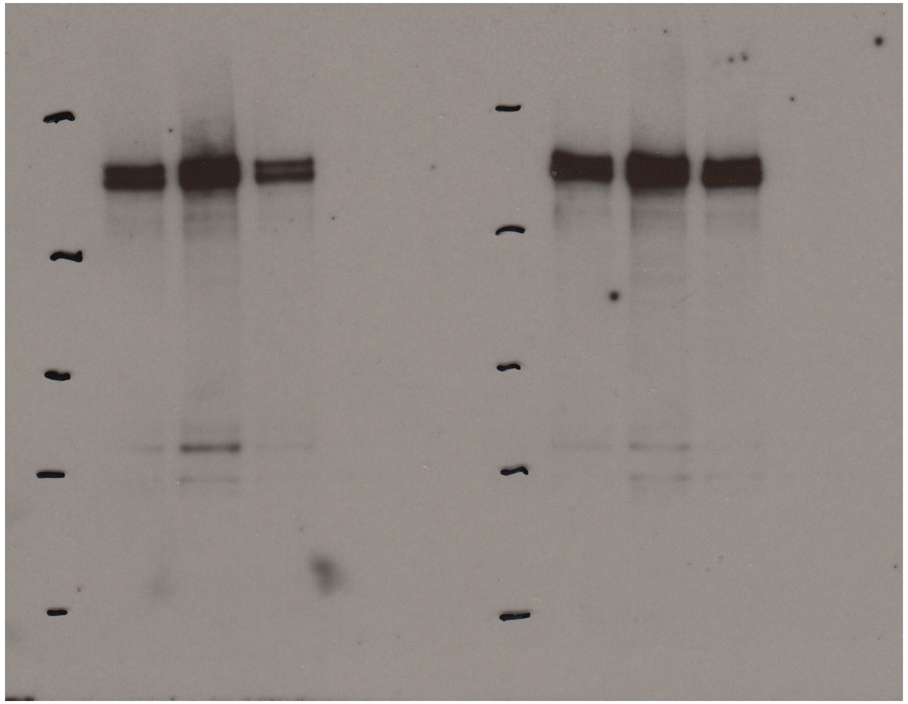

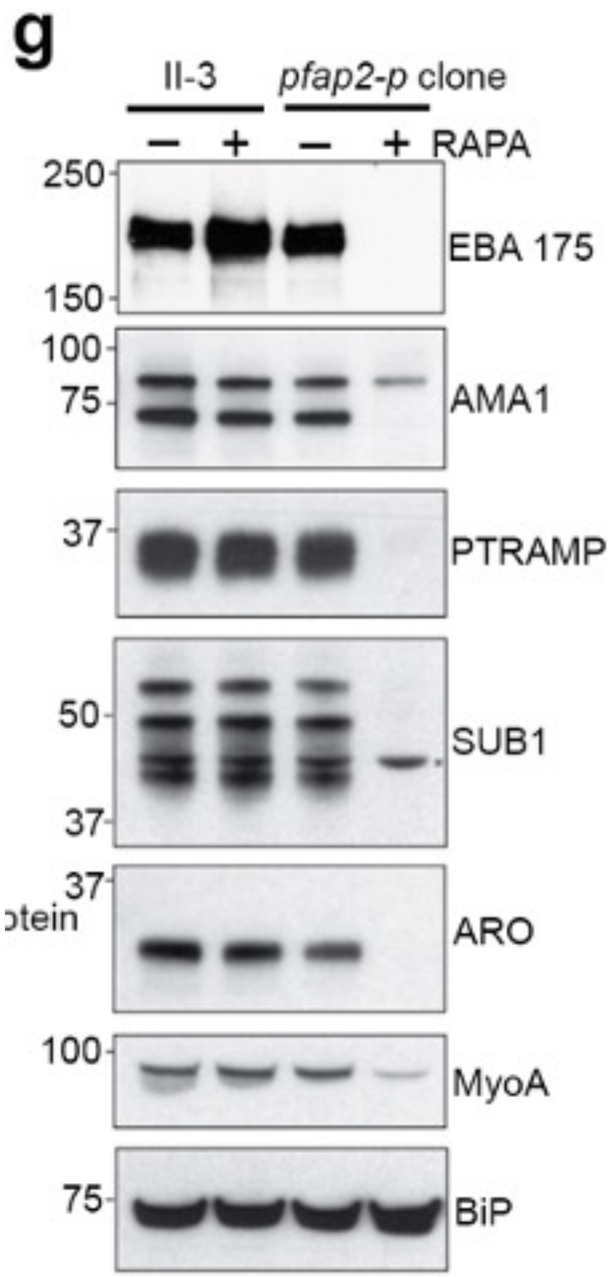

Fig. 2g

Uncropped image of PTRAMP

α -PTRAMP

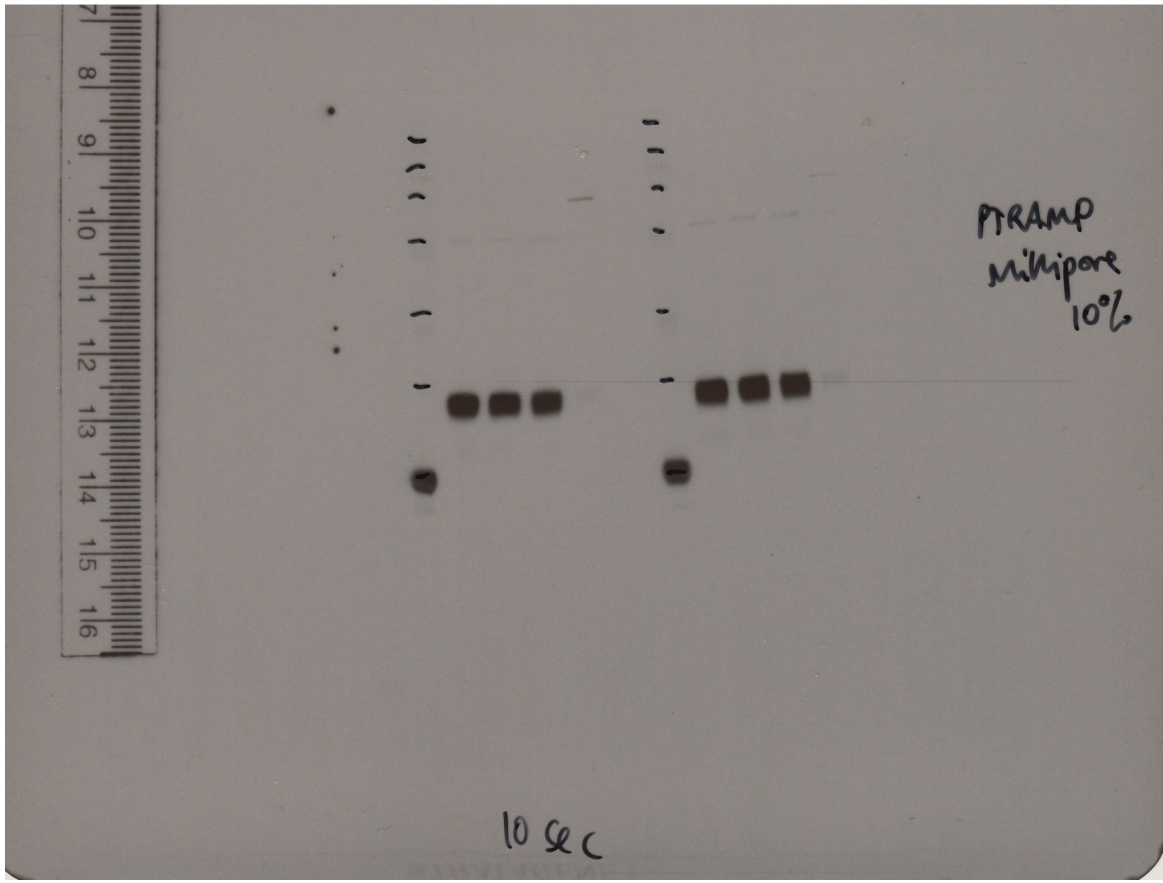

Fig. 2g

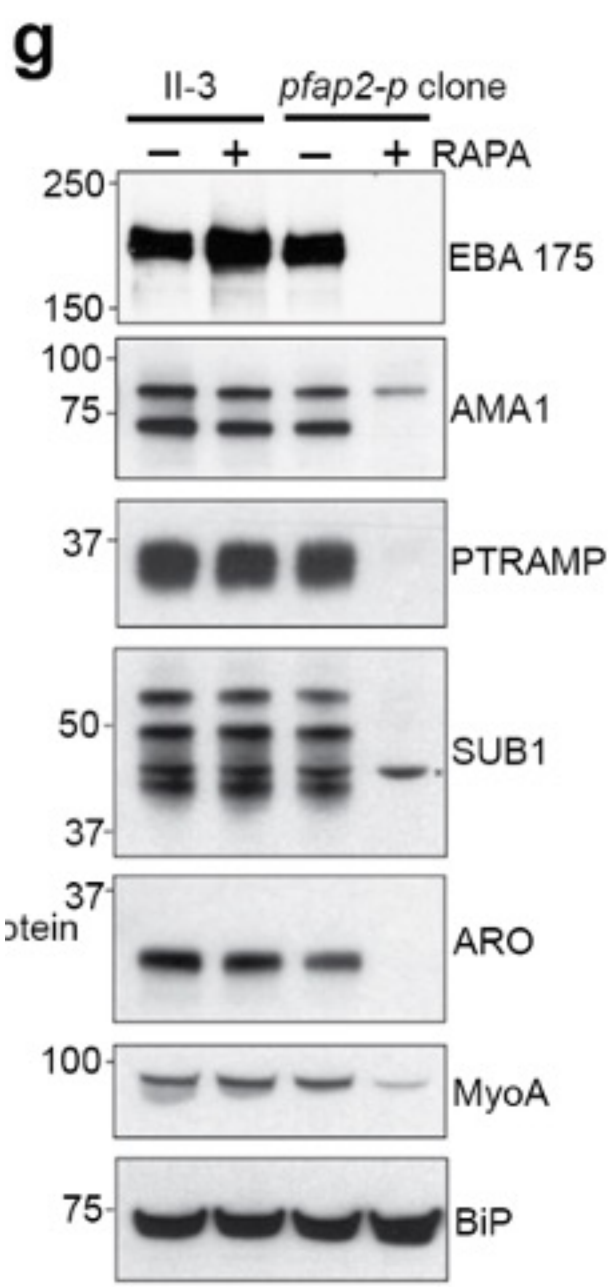

Uncropped image of SUB1

α -SUB1

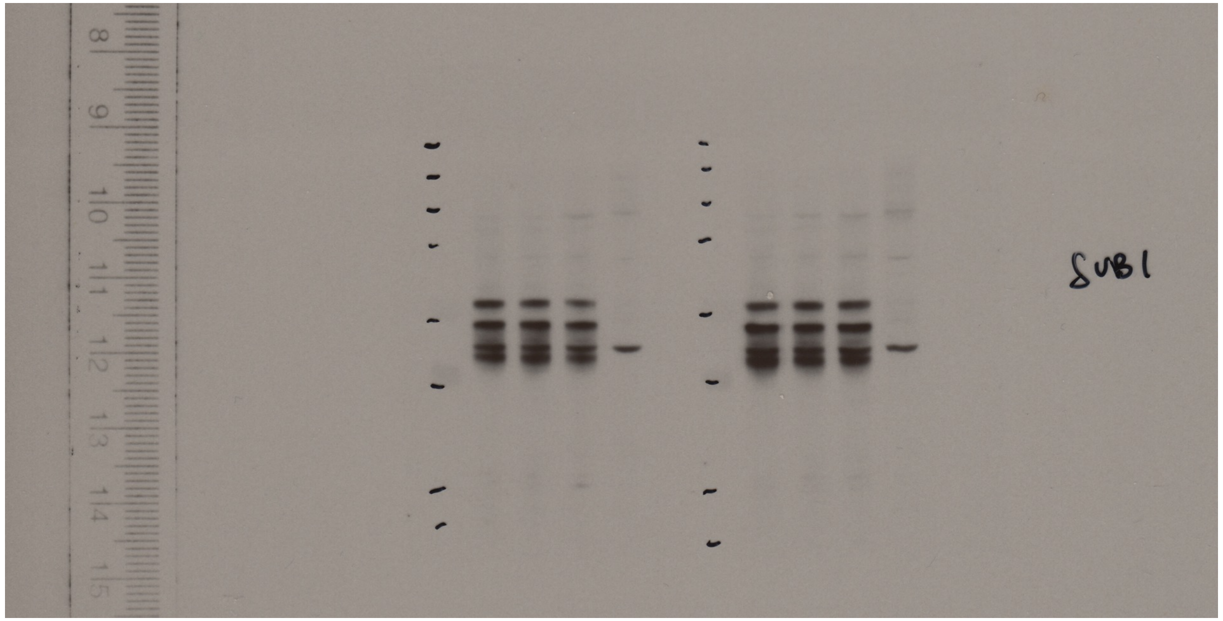

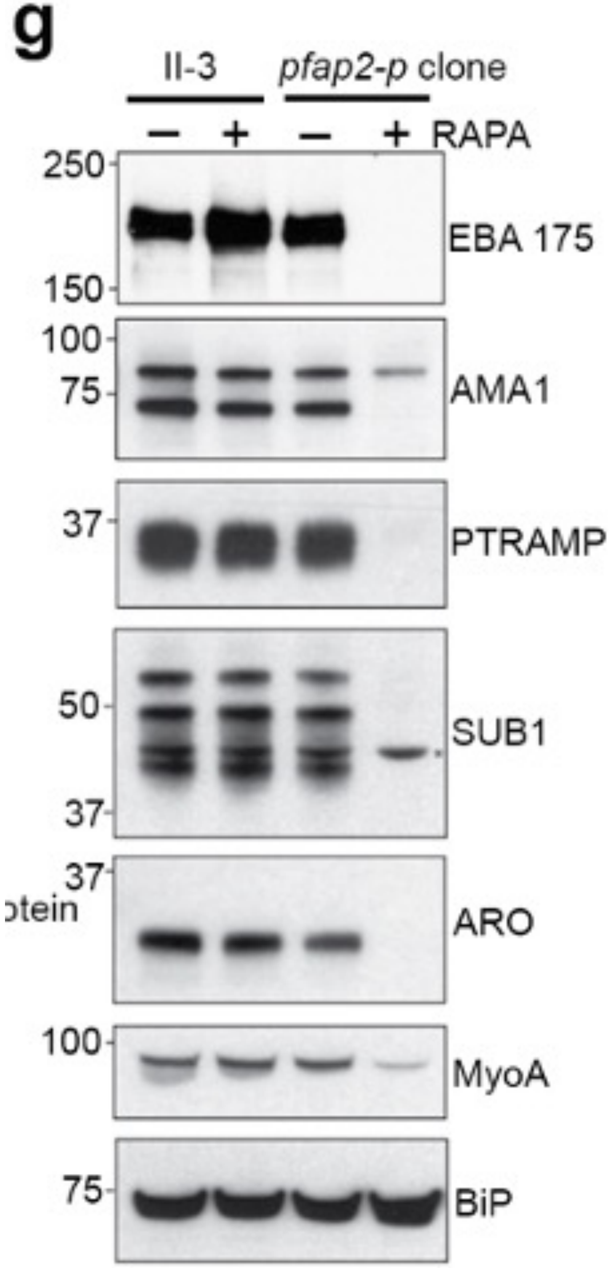

Fig. 2g

Uncropped image of AMA 1 and ARO

α -AMA1

α -ARO

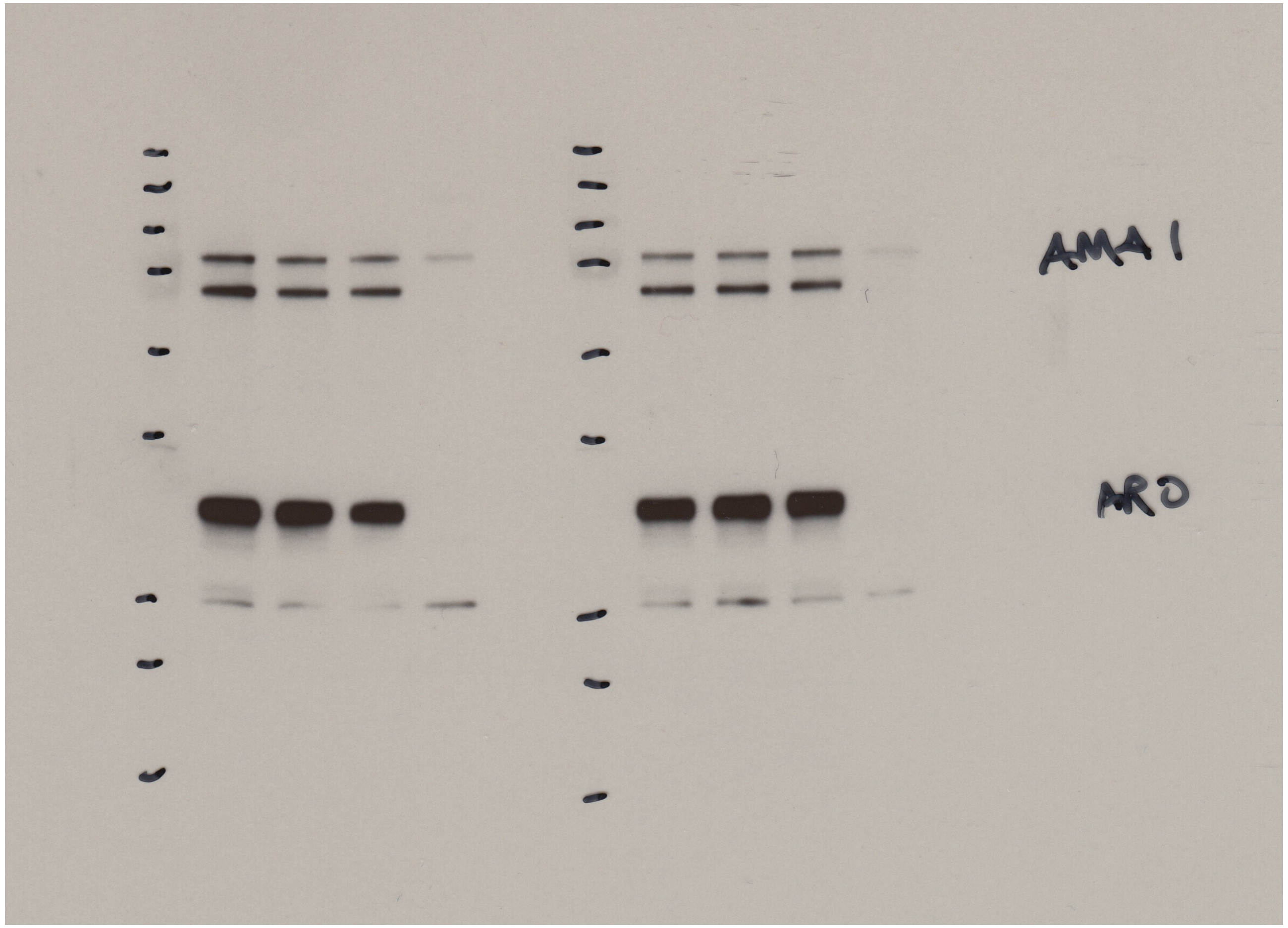

Fig. 2g

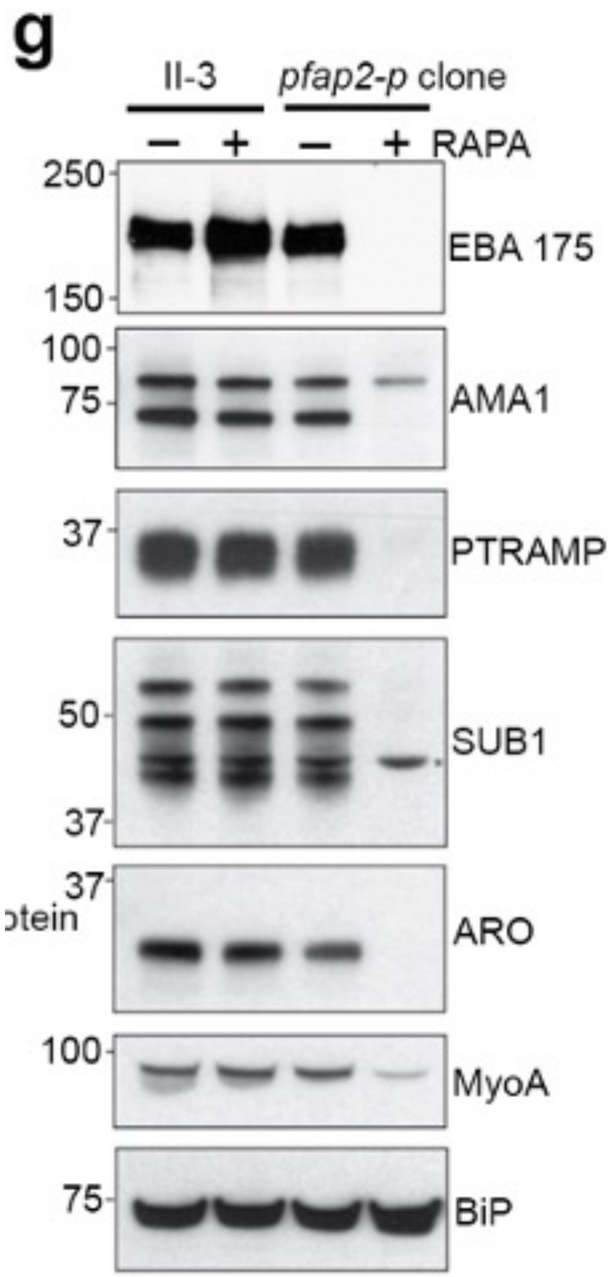

Uncropped image of MyoA

α -MyoA

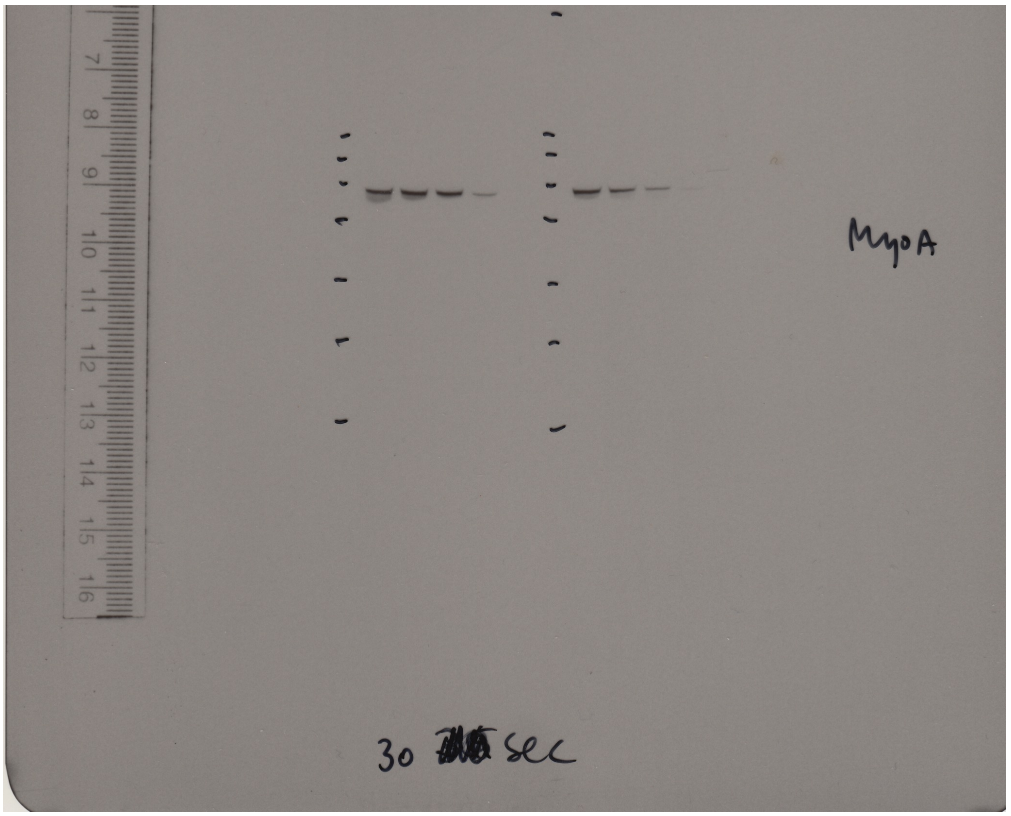

Fig. 2g

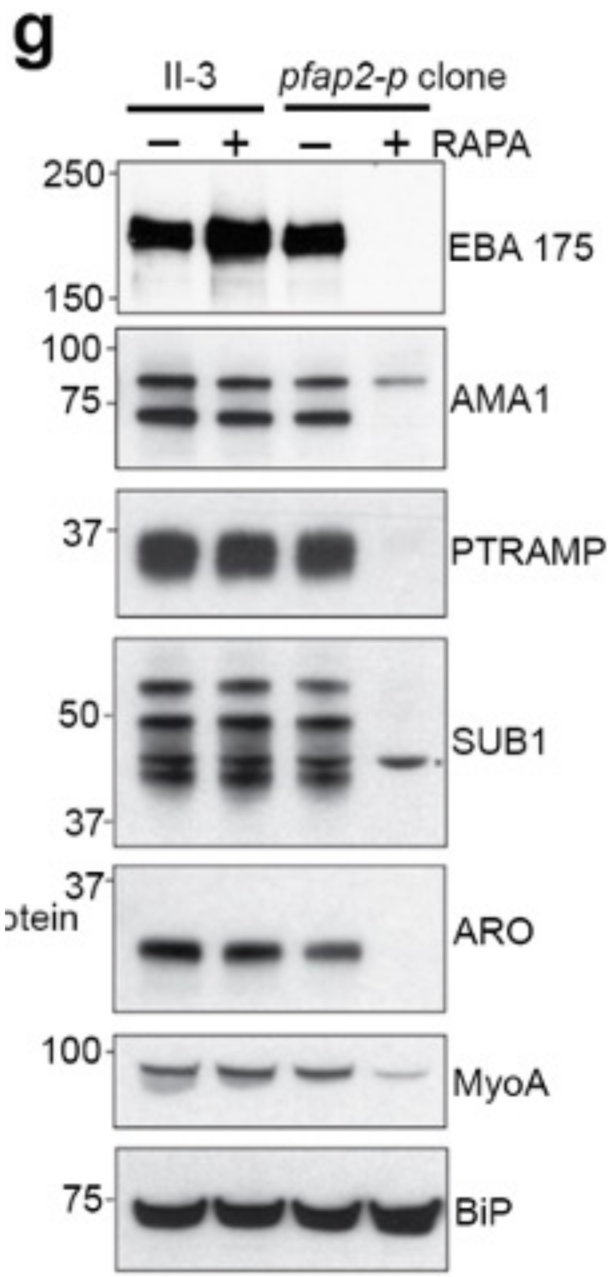

Uncropped image of BiP

α -BiP

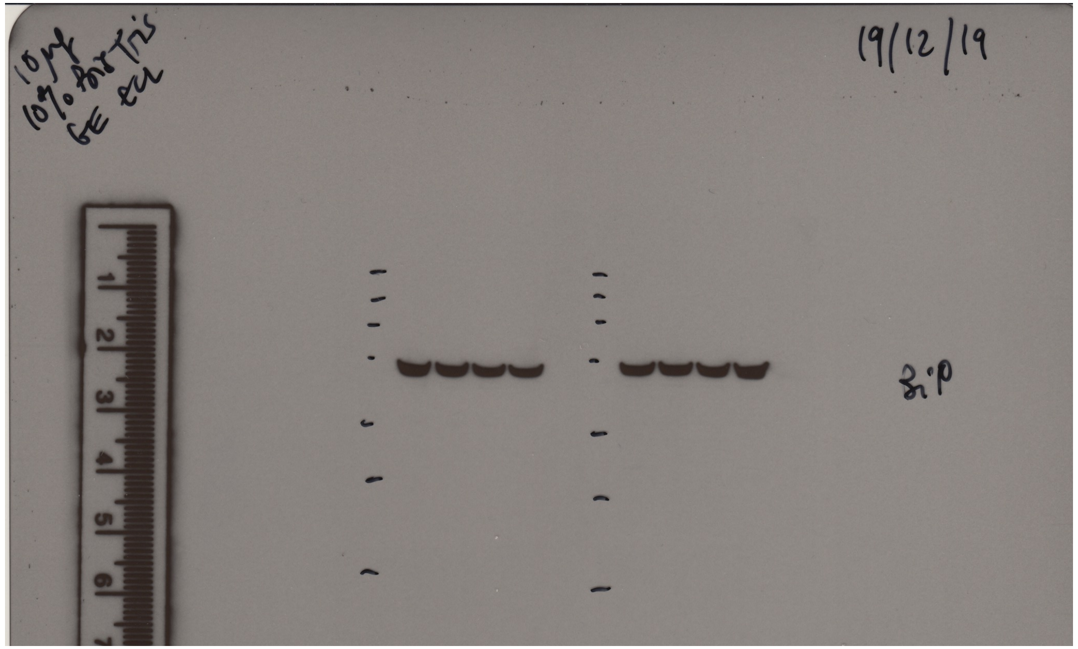

Supplement: Supplementary file 12 — Unprocessed western blots. [file 41564_2023_1497_MOESM12_ESM.pdf]
